# Supplementary material for: Uncomputability of phase diagrams
Source: Nat Commun. 2021 Jan 19;12:452. doi: 10.1038/s41467-020-20504-6 (PMC7815885; doi:10.1038/s41467-020-20504-6)
Supplement: Supplementary file 1 — Supplementary Information [file 41467_2020_20504_MOESM1_ESM.pdf]

Supplementary Information
Uncomputability of Phase Diagrams

Johannes Bausch*[1], Toby S. Cubitt†[2], and James D. Watson‡[2]

[1]CQIF, DAMTP, University of Cambridge, UK
[2]Department of Computer Science, University College London, UK

November 12, 2020

Supplementary Note 1 Preliminaries and Definitions

Gapped and gapless are rigorously defined as follows:

Supplementary Definition 1.1 (Gapped, from [CPGW15]). *We say that* $\mathbf{H}^{\Lambda(L)}$ *of Hamiltonians is gapped if there is a constant* $\gamma > 0$ *and a system size* $L_0 \in \mathbb{N}$ *such that for all* $L > L_0$, $\lambda_{\min}(\mathbf{H}^{\Lambda(L)})$ *is non-degenerate and* $\Delta(\mathbf{H}^{\Lambda(L)}) \geq \gamma$. *In this case, we say that* the spectral gap is at least γ.

Supplementary Definition 1.2 (Gapless, from [CPGW15]). *We say that* $\mathbf{H}^{\Lambda(L)}$ *is gapless if there is a constant* $c > 0$ *such that for all* $\epsilon > 0$ *there is an* $L_0 \in \mathbb{N}$ *so that for all* $L > L_0$ *any point in* $[\lambda_{\min}(\mathbf{H}^{\Lambda(L)}), \lambda_{\min}(\mathbf{H}^{\Lambda(L)}) + c]$ *is within distance* ϵ *from* $\operatorname{spec}\mathbf{H}^{\Lambda(L)}$.

As noted in [CPGW15], gapped is not defined as the negation of gapless; there are systems that fall into neither class, such as systems with closing gap or degenerate ground states. However, the stronger definitions allow us to avoid any potentially ambiguous cases.

Supplementary Definition 1.3 (Continuous family of Hamiltonians). *We say that a Hamiltonian* $\mathbf{H}(\varphi) = \sum_j \mathbf{h}_j(\varphi)$ *depending on a parameter* $\varphi \in \mathbb{R}$, *made up of a sum over local terms* $\mathbf{h}_j(\varphi)$ *each acting on a local Hilbert space* $\mathcal{H}$, *is* continuous *if each* $\mathbf{h}_j(\varphi) : \mathbb{R} \longrightarrow \mathcal{B}(\mathcal{H})$ *is a continuous function. We say that a family of Hamiltonians* $\{\mathbf{H}_i(\varphi)\}_{i\in I}$ *for some index set* I *is a* continuous family *if each* $\mathbf{H}_i(\varphi)$ *is continuous.*

Supplementary Note 2 Modified Quantum Phase Estimation

Here we discuss the modified version of quantum phase estimation which will be encoded in the Hamiltonian as discussed in the main body of the paper.

*jkrb2@cam.ac.uk
†t.cubitt@ucl.ac.uk
‡ucapjdj@ucl.ac.uk

The role of the quantum phase estimation (QPE) is simply to output φ in binary on the tape so that it can later be used as the input to a universal quantum TM. The idea will be to take a given unitary with eigenvalue $e^{i\pi\varphi}$ and its associated eigenvector, and then use QPE to provide an estimate of φ with the greatest precision possible. The precision of the QPE procedure will be limited by two factors: imperfect gates and the fact the binary length of φ may be longer than the available track length [NC10].

We first review the QPE used in the related works of [CPGW15] and [Bau+18b] before explaining how our QPE procedure is different and why it needs to be so. We characterise the amount of error the modified QPE procedure introduces which will play an important part in the construction of the Hamiltonian in later parts of the construction.

2.1 The State of the Art

In [CPGW15], the QPE on a unitary $\mathbf{U}_\varphi$ with eigenvalue $\mathrm{e}^{i\pi\varphi}$ can output the binary form of φ exactly: this is due to the fact that, in their construction, the QTM has access to a perfect gate set that is sufficient to expand exactly $|\varphi|$ digits—in particular, the standard QPE algorithm requires performing small controlled rotation gates $\mathbf{R}_n$ with angles $2^{i\pi 2^{-n}}$ for $n = 1, \dots, |\varphi|$, and since $|\varphi|$ is explicitly encoded in the local terms of the Hamiltonian, this circuit can be performed.

Furthermore, in [Bau+18b] the QPE procedure can also be performed perfectly by the same method. In addition one can detect when the binary expansion of φ is too long for the tape available to the QTM and penalize said segment lengths accordingly—the Marker Hamiltonian then has as a ground state which is a partition of the 1D spin chain into segments of length just long enough to perform QPE on φ and for the dovetailed TM to halt—if it halts.

In our new construction the situation is fundamentally different. Since the local terms of our Hamiltonian $\mathbf{H}^\Lambda(\varphi)$ do not explicitly depend on $|\varphi|$ anymore, we cannot provide the QPE with a set of rotation gates sufficient to perform an exact quantum Fourier transform (QFT). This means that we cannot guarantee the parameter we are estimating has a binary expansion short enough to be written on the tape available.

We therefore have to change the construction in two key ways. First, our encoding of φ will be in unary instead of binary. Since this is a undecidability result we are not constrained by poly-time reductions—or indeed any finite computational resources; any runtime overhead is acceptable. Secondly, we will perform some gates in the QPE only approximately. The gate approximation uses standard gate synthesis algorithms from Solovay-Kitaev [DN05a], where we gear the precision of the algorithm such that it suffices to obtain a large enough certainty on the first j digits of φ, given our tape has said length. The error resulting from truncating φ to j digit is more

involved, as QPE yields a superposition of states close *in value* to φ, which can for example mean that it rounds an expansion like 0.00001111 to 0.00010. We will circumvent this issue by choosing an encoding which lets us easily discover and penalize a too-short expansion, similar to the one in [Bau+18b].

2.2 Notation

Throughout we will denote the binary expansion of a number x as $\bar{x}$, and the first j digits of such an expansion as $\bar{x}_{\dots j}$. A questionmark ? will denote a digit that can either be a 0 or a 1. The jth digit of $\bar{x}$ will then be $\bar{x}_j$. For a given number x, we define $\operatorname{clz} x$ to be the count of leading zeros until the first 1 within $\bar{x}$—where we set $\operatorname{clz} 0 = \infty$. Similarly, we define the string $\operatorname{pfx} x$ to be the prefix of the string $\bar{x}$ such that $\operatorname{pfx} x = 0^{\times \operatorname{clz} x}1$, i.e. $\bar{x} = (\operatorname{pfx} x)??\dots$.

Within this section, we will further denote by $\mathbf{U}_\varphi$ a local unitary operator with eigenvalue $\mathrm{e}^{i\pi\varphi}$, and will refer to φ as the phase to be extracted.

Finally, let $\mathcal{M}$ be a universal reversible classical TM that takes its input in unary, i.e. as a string $00\dots0100\dots$; everything past the first leading one will be ignored; we lift $\mathcal{M}$ to a quantum TM by standard procedures [BV97].

In the following analysis we first start with an encoding scheme and analyse how the approximate QPE behaves on it; we finally show that each encoded parameter φ admits a small ϵ-ball around it where the system behaves in an identical fashion, making the behaviour of gapped vs. gapless robust and showing that our family of Hamiltonians is undecidable on a non-zero-measure set over the entire parameter range $\varphi \in [0, 1]$. We do not make a claim of knowing how the construction behaves for *any* choice of parameter. That is, given a particular value of φ, even if the halting behaviour of $\mathcal{M}$ on input $\operatorname{clz}\varphi$ were known, this would not always be sufficient to determine the behaviour of the Hamiltonian at this point.

2.3 Exact QPE with Truncated Expansion

We deal with the expansion error of our phase estimation first. As already mentioned, we need to choose an encoding that lets us detect and penalize expansion failure.

Supplementary Definition 2.1 (Unary Encoding)**.** *Let $\eta \in \mathbb{N}$ be the input we wish to encode. Then*

$$\varphi = \varphi(\eta) := 0.\underbrace{\mathsf{000}\cdots\mathsf{0}}_{\eta-1\ \text{digits}}\mathsf{100}\cdots \equiv 2^{-\eta}.$$

As mentioned, it is unclear a priori how much overlap the post-QPE state has with binary strings that encode the same number in unary (i.e. the string with the same number of leading 0 digits). The benefit of using the above encoding is that phase estimation tends to *round* numbers that are too short

to be expanded in full. Since we are encoding small numbers (assuming a little Endian bit order), this rounding will produce a large overlap with the all-zero state $|\bar{0}\rangle$. If we then penalize this outcome—e.g. by defining the dovetailed TM to move right forever on a zero input, which means it does not halt—we can ensure that the tape length will be extended until the input can be read in full, at which point there is no further expansion error to deal with.

As a first step we analyse the approximate quantum phase estimation procedure and compare the associated error with the perfect case, meaning that for now we give the QTM access to the same operations as in [CPGW15] and [Bau+18b], which includes access to the unitary $\mathbf{U}_\varphi$ and rotation gates $\mathbf{R}_n = \begin{pmatrix} 1 & 0 \\ 0 & 2^{i\pi 2^{-|\varphi|}} \end{pmatrix}$ which suffice to perform phase estimation exactly. We then do the QPE algorithm identically to that laid out in [CPGW15]; as this is the standard QPE algorithm from [NC10], we phrase the following lemma in a generic way.

Supplementary Lemma 2.2. *Let $\varphi(\eta) \in \mathbb{R}$ be a unary encoding of $\eta \in \mathbb{N}$ as per supplementary definition 2.1. On t qubits of precision, QPE is performed on the unitary $\mathbf{U}_\varphi$ encoding $\varphi(\eta)$ and its associated eigenstate defined in supplementary definition 2.1; denote the QPE output by $|\chi\rangle$. Then either:*

1. *$t \geq |\varphi|$, and $|\chi\rangle = |\bar{\varphi}\rangle$,*
2. *$t < |\varphi|$, and*
$$|\chi\rangle = \sum_{x \in \{0,1\}^t} \beta_x |x\rangle \quad \text{with} \quad |\beta_0| \geq \frac{1}{2}.$$

Proof. The first case is clear—we have a perfect gate set and sufficient tape, hence QPE is performed exactly [NC10].

For the second case where $t < |\varphi|$ the β_x are given in [NC10, eq. 5.25],

$$\beta_x = \frac{1}{2^t} \frac{1 - \exp(2\pi i(2^t\varphi - (b + x)))}{1 - \exp(2\pi i(\varphi - (b + x)/2^t))}, \tag{1}$$

where b is the best t bit approximation to φ *less* than φ, i.e. $0 \leq \varphi - 2^{-t}b \leq 2^{-t}$. Since we are using a unary encoding, as per supplementary definition 2.1, it is always the case that $b \equiv 0$, and therefore

$$\beta_x = \frac{1}{2^t} \frac{1 - \exp(2\pi i(2^{t-a} - x))}{1 - \exp(2\pi i(2^{-a} - x/2^t))}$$

Considering the case $x = b$ gives:

$$\begin{aligned}|\beta_0| &= \frac{1}{2^t}\frac{|1-\exp(2\pi i 2^{t-a})|}{|1-\exp(2\pi i 2^{-a})|} \\ &= \frac{1}{2^t}\frac{\sin(\pi 2^{t-a})}{\sin(\pi 2^{-a})} \\ &\geq \frac{1}{2^t}\frac{(\pi 2^{t-a})}{\pi 2^{-a}} = \frac{1}{2}.\end{aligned}$$

Going from the second last to last line we have used that $x/2 \leq \sin(x) \leq x$ for $x \in [0, \pi/2)$. □

Supplementary Corollary 2.3. *Take some $\eta \in \mathbb{N}$ and $\varphi(\eta)$ as defined in supplementary definition 2.1. Running the same quantum phase estimation QTM as in [CPGW15] to precision m bits yields an output state $|\chi\rangle$ given in supplementary lemma 2.2, such that either*

1. *$m \geq \eta$ and $|\chi\rangle = |\bar{\varphi}(\eta)\rangle$, or*

2. *$m < \eta$ and $|\langle\chi|0\rangle| \geq 1/2$.*

What if $\varphi(\eta)$ is not exactly given by the encoding in supplementary definition 2.1? It is clear that $|\chi\rangle$ is still a superposition of bit strings $|x\rangle$, weighted by β_x as in supplementary eq. (1). But our encoding allows us to derive a variant for supplementary corollary 2.3 that applies to an interval around the correctly-encoded inputs. Here we prove that we still have a large overlap with the all zero if the phase φ is not expanded fully.

Supplementary Corollary 2.4. *Let $\eta \in \mathbb{N}$, and $\varphi(\eta)$ as in supplementary definition 2.1. Take a perturbed phase $\varphi' \in [\varphi(\eta), \varphi(\eta) + 2^{-\eta-\ell})$ for some $\ell \in \mathbb{N}$, $\ell \geq 1$. Running the same quantum phase estimation QTM as in [CPGW15] to precision m bits yields an output state $|\chi\rangle$ given in supplementary lemma 2.2, such that either*

1. *$m \geq \eta$ and $|\langle\chi|\bar{\varphi}(\eta)\rangle| \geq 1 - 2^{-\ell}$, or*

2. *$m < \eta$, and $|\langle\chi|0\rangle| \geq 1/4$.*

Proof. We start with the first case. Take β_x from supplementary eq. (1). Assume for now that $m = \eta$; for increasing m the overlap with $\bar{\varphi}(\eta)$ can only increase. It is clear that the best m bit approximation to φ' less than φ' is given by $b = 2^m\bar{\varphi}(\eta)$ (as the first η digits of both are identical, and $\bar{\varphi}'_{\eta+1} = 0$ by assumption). Then

$$|\beta_0| = \frac{1}{2^m}\frac{|1-\exp(2\pi i(2^m\varphi' - b))|}{|1-\exp(2\pi i(\varphi' - b/2^m))|} = \frac{1}{2^m}\frac{\sin(\pi 2^m \epsilon)}{\sin(\pi\epsilon)} \geq 1 - 2^{-\ell},$$

where $\epsilon = \varphi' - b/2^m$, and the last inequality follows from $\sin(x)/x \geq 1 - x$ for $x \in [0, 1]$.

The second claim follows analogously: here again $b = 0$, and at most $2^m\varphi' \in [0, 3/4)$; the final bound is obtained by applying $x/4 \leq \sin(x) \leq x$ for $x \in [0, 3\pi/4)$, via

$$\frac{1}{2^m}\frac{\sin(\pi 2^m \varphi')}{\sin(\pi\varphi')} \geq \frac{1}{4}. \qquad \square$$

2.4 Solovay-Kitaev Modification to Phase Estimation

The second step in our QPE analysis is to approximate the small rotation gates that were previously allowed in supplementary corollary 2.3. We construct a QTM which only uses a standard gate set and $\mathbf{U}_\varphi$ for some $\varphi = \varphi(\eta) = 2^{-\eta}$, to run Quantum Phase Estimation (QPE) on $\mathbf{U}_\varphi$ and output a state which is very close in fidelity to the expansion of φ if done without error (i.e. if all gates were exact).

First note that all steps of the QPE procedure as described in [CPGW15] can be done exactly up to applying the phase gradient and locating the least significant bit—i.e. up until Section 3.6. However, after this, controlled rotation gates of the form $\mathbf{R}_n = 2^{i\pi 2^{-n}}$, for $1 \leq n \leq |\bar{\varphi}| = \eta$, need to be applied to perform the inverse QFT. In [CPGW15], this was done by further giving the QTM access to the gate $2^{i\pi 2^{-\eta}}$. To circumvent this necessity, we approximate small rotation gates using the Solovay-Kitaev algorithm.

2.4.1 Solovay-Kitaev QTM

First we introduce the standard statement for the existence of a TM which outputs a high precision approximation to the gate $\mathbf{R}_n = 2^{i\pi 2^{-\eta}}$ using the Solovay-Kitaev algorithm.

Supplementary Lemma 2.5 (SK Machine [DN05b]). *There exists a classical TM which, given an integer k and maximum error ϵ, outputs an approximation $\tilde{\mathbf{R}}_k$ to the gate $\mathbf{R}_k \in \mathrm{SU}(2)$ such that $\|\tilde{\mathbf{R}}_k - \mathbf{R}_k\| < \epsilon$. The TM runs in time and space $\mathrm{O}(\log^{c_1}(1/\epsilon))$ for some $3.97 < c_1 < 4$.*

Part way through the quantum phase estimation procedure, we need to apply the inverse QFT. However, we do not have access to gates of the form $2^{i\pi 2^{-\eta}}$, and our entire QTM will be limited to space L. As a result, whenever the procedure requires a $2^{i\pi 2^{-\eta}}$-gate or a power of such a gate, we run the Solovay-Kitaev algorithm to generate an approximation. As there is $\mathrm{O}(\eta^2)$ many gates to be approximated overall, the procedure will have to be repeated this many times.

However, since we are performing the QPE on a finite length tape, we only have L qubits onto which we can write out the output of the Solovay-Kitaev algorithm; this limits the precision we can achieve using this technique.

Inverting the space bound in supplementary lemma 2.5 with respect to the error ϵ, the best approximation obtainable is thus

$$\left\|\tilde{\mathbf{R}}_k - \mathbf{R}_k\right\| \le \mathrm{e}^{-\,\mathrm{O}(L^{1/c_1})} \le 2^{-c_2 L^{1/c_1}}, \tag{2}$$

where we wrote the constant in the exponent as c_2. Both Solovay-Kitaev constants c_1 and c_2 can be written down explicitly.

2.4.2 Approximation Error for Output State

The gates used in the inverse QFT in the previous section were only performed up to a finite precision and hence there will be an error associated with the output state relative to the case with perfect gates. We will see that the output is then a state that is exponentially close to what would be expected in the case with perfect gates.

Let $\tilde{\mathbf{R}}_n$ be the approximation to the rotation gate $\mathbf{R}_n = 2^{\pi i 2^{-\eta}}$ such that $\|\tilde{\mathbf{R}}_n - \mathbf{R}_n\| < \epsilon$, where $\epsilon = 2^{-c_2 L^{1/c_1}}$ is given by the Solovay-Kitaev theorem, supplementary eq. (2) and supplementary lemma 2.5.

Supplementary Lemma 2.6. *Let $\mathbf{U}_{\mathrm{QPE}}$ be the unitary describing the implementation of QPE by a QTM on m qubits with each gate performed exactly. Let $\tilde{\mathbf{U}}_{\mathrm{QPE}}$ be the unitary describing the same QPE algorithm on m qubits, but where Solovay-Kitaev is used to approximate the rotation gates $\mathbf{R}_n$ to precision ϵ; all other gates are implemented exactly. Then the total error of the approximate QPE is*

$$\left\|\tilde{\mathbf{U}}_{\mathrm{QPE}} - \mathbf{U}_{\mathrm{QPE}}\right\| < \frac{m^2}{2}\epsilon = \frac{m^2}{2} 2^{-c_2 L^{1/c_1}}. \tag{3}$$

Proof. The first part of the phase estimation procedure—the phase gradient operations $\mathbf{U}_{\mathrm{PG}}$—can be done exactly in both the approximate and exact cases. If QPE is performed to m qudits, we see that there are $m^2/2$ applications of $\mathbf{R}_n$ gates during the inverse QFT procedure. As $\tilde{\mathbf{U}}_{\mathrm{QPE}} = \tilde{\mathbf{U}}^\dagger_{\mathrm{QFT}} \mathbf{U}_{\mathrm{PG}}$, the claim follows from applying the triangle inequality $m^2/2$ times. □

2.5 Total Quantum Phase Estimation Error

We have seen previously that there will be errors from both the fact that the parameter φ may have a binary expansion longer than the tape length available, and from the Solovay-Kitaev (S-K) algorithm we use to approximate and apply the rotation gates. Here we combine the two errors and upper bound the total deviation introduced. We continue using m to denote the number of binary digits that φ is expanded to, and L is the full tape length.

We emphasize that the two are not necessarily identical, as we can always cordon off a section of the tape to restrict the QPE to only work to within a more limited precision—i.e. we can execute the QPE TM on a subsegment

of size $m \leq L$ as in supplementary corollary 2.3, and approximate the latter with Solovay-Kitaev that itself can make use of the full tape space available, i.e. L. For now we treat L and m as independent quantities, regardless of how they are implemented, and we will choose their specific relation in due course.

Supplementary Lemma 2.7. *Let $\eta \in \mathbb{N}$ and $\varphi(\eta) \in \mathbb{R}$ as in supplementary definition 2.1, and take $\tilde{\mathbf{U}}_{\mathrm{QPE}}$ as the Solovay-Kitaev QPE unitary with output $|\tilde{\chi}\rangle$. Then either*

1. *$m \geq \eta$ and $|\langle\tilde{\chi}|\bar{\varphi}(\eta)\rangle| \geq 1 - \delta(L,m)$, or*
2. *$m < \eta$ and $|\langle\tilde{\chi}|0\rangle| \geq 1/2 - \delta(L,m)$.*

Here

$$\delta(L,m) < \frac{m^2}{2} 2^{-c_2 L^{1/c_1}}.$$

Proof. Immediate from supplementary lemma 2.6, supplementary eq. (2), and supplementary corollary 2.3. □

As before, we add an approximate variant for the case where $\varphi' \neq \varphi(\eta)$.

Supplementary Lemma 2.8. *Let $\eta \in \mathbb{N}$, and $\varphi(\eta)$ as in supplementary definition 2.1. Take a perturbed phase $\varphi' \in [\varphi(\eta), \varphi(\eta) + 2^{-\eta-\ell})$ for $\ell \in \mathbb{N}$, $\ell \geq 1$, and consider the same setup as in supplementary lemma 2.7. Then either*

1. *$m \geq \eta$ and $|\langle\tilde{\chi}|\bar{\varphi}(\eta)\rangle| \geq 1 - 2^{-\ell} - \delta(L,m)$, or*
2. *$m < \eta$, and $|\langle\tilde{\chi}|0\rangle| \geq 1/4 - \delta(L,m)$.*

Proof. Analogously to supplementary lemma 2.7, but using supplementary corollary 2.4. □

The bound in terms of $\delta(L,m)$ is only useful for large L, in which case it is easy to see that since $m \leq L$, $\delta \to 0$ for $L \to 0$. Since we need δ to be small in due course, we capture a more precise bound in the following remark.

Supplementary Remark 2.9. *For any $\delta_0 > 0$ there exists an $L_0 = L_0(c_1, c_2, \delta_0)$ such that $\delta(L,m) < \delta_0$ for all $L \geq L_0$, where $\delta(L,m)$ is defined in supplementary lemma 2.7, and c_1, c_2 are the Solovay-Kitaev constants from supplementary eq. (2).*

Proof. Clear. □

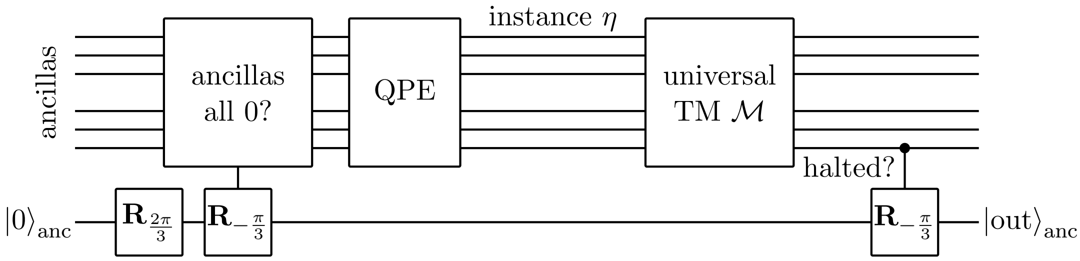

Supplementary Figure 1: QPE and universal TM circuit. The construction uses one flag ancilla $|0\rangle_{\rm anc}$ to verify that as many ancillas as necessary for the successive computation are correctly-initialized ancillas (e.g. $|0\rangle$), and if not rotating the single guaranteed $|0\rangle_{\rm anc}$ flag by $\pi/3$. On some ancillas, the problem instance l is written out. Another rotation by $\pi/3$ is applied depending on whether the dovetailed universal TM $\mathcal{M}$ halts on η or not within the number of steps allowed by the clock driving its execution, which in turn is limited by the tape length.

Supplementary Note 3 QPE and Universal QTM Hamiltonian

In this section we examine how to encode the quantum Turing machine performing quantum phase estimation described in supplementary note 2 (and briefly overviewed in section 4.2 of the main article) into a Hamiltonian on a spin chain of length L, such that the ground state energy of the Hamiltonian is non-negative if and only if a dovetailed universal Turing machine $\mathcal{M}$ halts on input $\varphi(\eta)$ and within tape length L. We further prove that this ground state energy remains non-negative (or negative, respectively) if instead of $\varphi(\eta)$ we are given a slightly perturbed phase $\varphi' \in [\varphi(\eta), \varphi(\eta) + 2^{-\eta-\ell})$, given $\ell \geq 1$ is large enough. This section makes rigorous the results at the end of section 4.2 of the main article.

We note that the circuit-to-Hamiltonin mapping used in this work will be a variation of that in [CPGW15], which is itself a modification of the construction in [GI09]. [GI09] is a particularly important work to to the field of Hamiltonian complexity as it demonstrates how a computation can be encoded in a 1D, nearest neighbour, translationally invariant Hamiltonian. This is particularily suprising as it shows the simplest class of Hamiltonians (in terms of dimensionality and interaction type) have hard to compute low-energy properties. Furthermore, classically this class of Hamiltonians is computationally tractable.

With the aim of proving the above statements about the ground state energy, we first amend the computation slightly. In [CPGW15], the authors used Gottesman and Irani's history state construction for a Turing machine with an initially empty tape [GI09]. To ensure a correctly initialised tape,

the authors use an initialization sweep; essentially a single sweep over the entire tape with a special head symbol, under which one can penalize a tape in the wrong state.

Instead of using an initialization sweep, we make do with a single ancilla (denoted with subscript "anc" in the following) which is initialized to $|0\rangle_{\mathrm{anc}}$, and verify on a circuit level that all the other ancillas are correctly initialized. In order to achieve this, we first execute a single $\mathbf{R}_{2\pi/3}$ rotation on $|0\rangle_{\mathrm{anc}}$ to initialize it to a $\mathbf{R}_{2\pi/3}\,|0\rangle_{\mathrm{anc}}$-rotated state. Next, we execute a controlled $\mathbf{R}_{-\pi/3}$ rotation in the opposite direction on $|\phi\rangle_{\mathrm{anc}}$, where the controls are on all the ancillas we wish to ensure are in the right state. If and only if *all* of the controlling ancillas are in state $|1\rangle$—which we can check e.g. with a multi-anticontrolled operation—will we perform a rotation by $\mathbf{R}_{-\pi/3}$. After the controlled rotation, we apply $\mathbf{X}$ flips to all the ancillas we wish to initialize to $|0\rangle$.

This ancilla will carry another role: in case the dovetailed universal TM $\mathcal{M}$ from supplementary note 2 halts, we transition to a finalisation routine that performs another $\mathbf{R}_{-\pi/3}$ rotation on it. The net effect of this circuit is that, after the entire computation ends, the ancilla is in state $|\mathrm{out}\rangle_{\mathrm{anc}}$ with overlap

$$\langle 1|\mathrm{out}\rangle_{\mathrm{anc}} = \begin{cases} 0 & \text{if all ancillas are correctly initialized } \textit{and } \mathcal{M} \text{ halted, or} \\ \frac{\sqrt{3}}{2} & \text{otherwise.} \end{cases} \tag{4}$$

This idea in the context of circuit-to-Hamiltonian mappings was introduced in [BCO17]; for completeness we give an overall circuit diagram of the entire computation to be mapped to a Hamiltonian in supplementary fig. 1. We remark that breaking down a multi-controlled quantum gate into a local gate set is a standard procedure described e.g. in [NC10].

We formalise the above procedure in the following lemma:

Supplementary Lemma 3.1. *Consider an initial state*

$$|\psi_0\rangle = |0\rangle_{\mathrm{anc}} \left(\alpha\,|1\rangle^{\otimes L} + \sqrt{1-\alpha^2}\,|\phi\rangle\right) \quad \textit{where} \quad |\phi\rangle \perp |1\rangle^{\otimes L}.$$

Assume the Turing machine $\mathcal{M}$ halts with probability ϵ when acting on an initial state $|0\rangle_{\mathrm{anc}}\,|1\rangle^{\otimes L}$. Then, the final output state of the computation $|\psi_T\rangle$ satisfies

$$\langle\psi_T|\left[|1\rangle\langle 1|_{\mathrm{anc}} \otimes \mathbb{1}^{\otimes L}\right]|\psi_T\rangle = \frac{3}{4}\left(1-\alpha^2\epsilon^2\right).$$

Proof. By explicit calculation, we have

$$
\begin{aligned}
|\psi_0\rangle &\xmapsto{\mathbf{R}_{2\pi/3}} \mathbf{R}_{2\pi/3}\,|0\rangle_{\text{anc}}\left(\alpha\,|1\rangle^{\otimes L} + \sqrt{1-\alpha^2}\,|\phi\rangle\right) \\
&\xmapsto{\mathbf{cR}_{-\pi/3}} \alpha\mathbf{R}_{\pi/3}\,|0\rangle_{\text{anc}}\,|1\rangle^{\otimes L} + \sqrt{1-\alpha^2}\mathbf{R}_{2\pi/3}\,|0\rangle\,|\phi\rangle \\
&\xrightarrow{\mathcal{M}} \alpha\mathbf{R}_{\pi/3}\,|0\rangle_{\text{anc}}\left(\epsilon\,|\psi_{\text{halt}}\rangle + \sqrt{1-\epsilon^2}\,|\psi_{\text{non-halt}}\rangle\right) \\
&\quad + \sqrt{1-\alpha^2}\mathbf{R}_{2\pi/3}\,|0\rangle_{\text{anc}}\left(\epsilon'\,|\phi_{\text{halt}}\rangle + \sqrt{1-\epsilon'^2}\,|\phi_{\text{non-halt}}\rangle\right) \\
&\xrightarrow{\mathbf{cR}_{-\pi/3}} \alpha\epsilon\,|0\rangle\,|\psi_{\text{halt}}\rangle + \alpha\sqrt{1-\epsilon^2}\mathbf{R}_{\pi/3}\,|0\rangle\,|\psi_{\text{non-halt}}\rangle \\
&\quad + \epsilon'\sqrt{1-\alpha^2}\mathbf{R}_{\pi/3}\,|0\rangle_{\text{anc}}\,|\phi_{\text{halt}}\rangle + \sqrt{1-\epsilon'^2}\sqrt{1-\alpha^2}\mathbf{R}_{2\pi/3}\,|0\rangle_{\text{anc}}\,|\phi_{\text{non-halt}}\rangle \\
&= |\psi_T\rangle\,.
\end{aligned}
$$

Using

$$
|0\rangle \xmapsto{\mathbf{R}_{\pi/3}} \cos\left(\frac{\pi}{3}\right)|0\rangle + \sin\left(\frac{\pi}{3}\right)|1\rangle \quad \text{and} \quad |0\rangle \xmapsto{\mathbf{R}_{2\pi/3}} \cos\left(\frac{\pi}{3}\right)|0\rangle - \sin\left(\frac{\pi}{3}\right)|1\rangle
$$

this means that

$$
\langle\psi_T|\left[|1\rangle\langle 1|_{\text{anc}} \otimes \mathbb{1}^{\otimes L}\right]|\psi_T\rangle = \sin^2\left(\frac{\pi}{3}\right)\left(1-\alpha^2\epsilon^2\right). \qquad \square
$$

3.1 Feynman-Kitaev Hamiltonian

Given our quantum Turing machine from supplementary note 2 augmented with a single necessary "good" ancilla $|0\rangle_{\text{anc}}$ as just described, we apply the Gottesman and Irani construction from [GI09] to translate our desired computation in the ground state of a one-dimensional, nearest neighbour, translationally invariant Hamiltonian with open boundary conditions. We summarize the core ideas to set up the notation used in this section, but refer the reader to [GI09; CPGW15; BC18] for details.

Supplementary Definition 3.2 (History state)**.** *A* history state $|\Psi\rangle \in \mathcal{H}_C \otimes \mathcal{H}_Q$ *is a quantum state of the form*

$$
|\Psi\rangle = \frac{1}{\sqrt{T}}\sum_{t=1}^{T}|t\rangle_C\,|\psi_t\rangle_Q\,, \tag{5}
$$

where $\{|1\rangle,\ldots,|T\rangle\}$ *is an orthonormal basis for* $\mathcal{H}_C$*, and* $|\psi_t\rangle = \prod_{i=1}^{t}\mathbf{U}_i\,|\psi_0\rangle$ *for some initial state* $|\psi_0\rangle \in \mathcal{H}_Q$ *and set of unitaries* $\mathbf{U}_i \in \mathcal{B}(\mathcal{H}_Q)$*.*

$\mathcal{H}_C$ *is called the* clock register *and* $\mathcal{H}_Q$ *is called the* computational register*. If* $\mathbf{U}_t$ *is the unitary transformation corresponding the* t^{th} *step of a quantum computation—which in our case is not a gate in the circuit model, but a QTM transition—then* $|\psi_t\rangle$ *is the state of the computation after* t *steps. We say that the history state* $|\Psi\rangle$ encodes *the evolution of the quantum computation.*

As discussed in supplementary note 2.5, the QPE Turing machine we devised has two meta parameters L and m. On a spin chain of length L, instead of expanding $L-3$ digits of φ as is the case in [CPGW15], we allow the expansion to happen on a smaller sub-segment of length m of the chain. This can be done dynamically, i.e. by adding a Turing machine before the QPE invocation which sections off a part $m = m(L)$ of the tape and places a distinct symbol $\textcircled{\text{II}}$ there. Since it is obvious how to do this we will not go into detail here, and remark that in the final construction we will choose $m = L-3$: an explicit construction for such a Turing machine is given in[Bau+18b, Lem. 15]. The QPE and dovetailed universal TM—augmented by the single-ancilla construction described at the start of this section—we will jointly call $\mathcal{M}' = \mathcal{M}'(L, m)$, i.e. such that there is L tape available; we emphasize that $\mathcal{M}'(L, m)$ has an identical set of symbols and internal states for all L and m.

In all of the following we will analyse the spectrum of the history state Hamiltonian within a "good" type of subspace, by which we mean a tape bounded by special endpoint states $\textcircled{<}$ and $\textcircled{>}$. This subspace will, analogous to the 2D undecidability construction, be called *bracketed* states; on an overall local Hilbert space $\mathcal{H} = \mathcal{H}_a \oplus \mathcal{H}_b$ such that $|\textcircled{<}\rangle, |\textcircled{>}\rangle \in \mathcal{H}_b$, we set

$$\mathcal{S}_{\rm br}(m) := |\textcircled{<}\rangle \otimes \mathcal{H}_a^{\otimes L} \otimes |\textcircled{>}\rangle . \tag{6}$$

Since no transition rule for the history state Hamiltonian ever moves these boundary markers, the overall Hamiltonian we construct will be block-diagonal with respect to signatures determined by the brackets. A standard argument then shows that within this bracketed subspace, the history state Hamiltonian encoding the QPE Turing machine behaves as designed, and we can analyse the spectrum therein by analysing the encoded computation. Outside of the bracketed subspace, a variant of the Clairvoyance lemma allows us to always lower-bound the energy, such that it does not interfere with the rest of the construction.

In order to make all of this precise, we first define the full QPE history state Hamiltonian in the following theorem, which is adapted from [CPGW15, Th. 10].

Theorem 3.3 (QPE history state Hamiltonian). *Let $L, m \in \mathbb{N}$, $0 < m \leq L-3$. Let there exist a Hermitian operator $\mathbf{h} \in \mathcal{B}(\mathbb{C}^d \otimes \mathbb{C}^d)$, where the local Hilbert space contains special marker states $|\textcircled{<}\rangle$ and $|\textcircled{>}\rangle$ that define the bracketed subspace $\mathcal{S}_{\rm br}$ as in supplementary eq. (6), such that*

1. $\mathbf{h} \geq 0$,
2. *d depends (at most polynomially) on the alphabet size and number of internal states of $\mathcal{M}'$,*
3. $\mathbf{h} = \mathbf{A} + \mathrm{e}^{i\pi\varphi(\eta)}\mathbf{B} + \mathrm{e}^{-i\pi\varphi(\eta)}\mathbf{B}^\dagger$, *where*

- $\mathbf{B} \in \mathcal{B}(\mathbb{C}^d \otimes \mathbb{C}^d)$ *independent of* η *and with coefficients in* $\mathbb{Z}$*, and*
- $\mathbf{A} \in \mathcal{B}(\mathbb{C}^d \otimes \mathbb{C}^d)$ *is Hermitian, independent of* η*, and with coefficients in* $\mathbb{Z} + \mathbb{Z}/\sqrt{2}$*;*

Furthermore, a spin chain of length L with local dimension d, the translationally-invariant nearest-neighbour Hamiltonian $\mathbf{H}_{\mathrm{QTM}}(L) := \sum_{i=1}^{L-1} \mathbf{h}^{(i,i+1)}$ *has the following properties.*

4. $\mathbf{H}_{\mathrm{QTM}}(L)$ *is frustration-free, and*

5. *the unique ground state of* $\mathbf{H}_{\mathrm{QTM}}(L)|_{\mathcal{S}_{\mathrm{br}}(m)}$ *is a computational history state as in supplementary definition 3.2 encoding the evolution of* $\mathcal{M}'(L, m)$*.*

The history state satisfies

6. $T = \Omega(\mathrm{poly}(L)2^L)$ *time-steps, in either the halting or non-halting case;*

7. *If* $\mathcal{M}'$ *runs out of tape within a time* T *less than the number of possible TM steps allowed by the history state clock, the computational history state only encodes the evolution of* $\mathcal{M}'$ *up to time* T*.*

8. *In either the halting or non-halting case, the remaining time steps of the evolution encoded in the history state leave the computational tape for* $\mathcal{M}'$ *unaltered, and instead the QTM runs an arbitary computation on a waste tape as described in [CPGW15].*

Proof. Almost all of the above follows from [CPGW15, Th. 10]. Item 3 differs only in that we have removed any dependence on $e^{i\pi 2^{-|\varphi|}}$ due to the new modified transition rules, as we now approximate the necessary rotations using the Solovay-Kitaev theorem (see supplementary note 2). □

3.1.1 Clock Construction

The history state Hamiltonian described above encodes an evolution of a computation for $T(L)$ steps, where $T(L)$ *does not* depend on the computation itself. This ensures that the history state will be a superposition over $T(L)$ time steps independent on whether $\mathcal{M}'$ halts on the tape of length $L-2$ and with cordonned-off subsection m. As mentioned previously, in the case of the computation halting, this is done by forcing the QTM head to switch to an additional "waste tape" where an arbitrary computation is performed until the clock finishes.

Supplementary Theorem 3.3 uses the clock construction designed in [CPGW15, sec. 4.2, 4.3, 4.4]. Bounds on the clock runtime are readily obtained: if $T(L)$ denotes the runtime of the clock on a tape of length L, we have

$$\Omega\left(L\xi^L\right) \leq T(L) \leq \mathrm{O}\left(L\xi^L \log(L)\right) \tag{7}$$

for some constant $\xi \in \mathbb{N}$.

3.1.2 QTM and Clock Combined

Supplementary Theorem 3.3 combines the QTM and clock such that the QTM head only makes a transition when the oscillator from the clock part of the history state passes over the QTM head. Details can be found in [CPGW15, sec. 4.6.1].

3.2 The Initialisation and Non-Halting Penalty

We now want to introduce a penalty term which will penalise computations that have not halted and not been initialised correctly.

Initialisation Penalty. In order to ensure that the single ancilla we require is correctly initialized, we introduce a projector that penalizes $|\psi\rangle_{\mathrm{anc}}$ in any state but $|0\rangle_{\mathrm{anc}}$ at the start of the computation. This can be done by a term of the form $|0\rangle\langle 0|_{\mathrm{C}} \otimes (\mathbb{1} - |1\rangle\langle 1|)_{\mathrm{anc}}$, which is local if and only if we can locally detect the initial clock state $|0\rangle_{\mathrm{C}}$ above the single ancilla on the tape. As per the constructions in [GI09; CPGW15], this state can indeed be locally detected.

Finalisation Penalty. The final penalty follows precisely the same pattern: we add a local projector of the form $|T\rangle\langle T|_{\mathrm{C}} \otimes (\mathbb{1} - |1\rangle\langle 1|)_{\mathrm{anc}}$, and ensure that the final clock state $|T\rangle_{\mathrm{C}}$ can be recognized locally above where $|\mathrm{out}\rangle_{\mathrm{anc}}$ sits. To realise this, we note that the ancilla bit is located at the end of string of qudits encoding the TM tape. The final clock state can then be locally determined by a nearest-neighbour, translationally invariant term that recognises the final clock state by looking at the pair of qudits at the end of the chain. Again, this is done in [GI09; CPGW15].

Penalty Term Construction. The amplitude of the output ancilla $|\psi\rangle_{\mathrm{anc}}$ depends on correct initialization of the ancillas for the QTM, as well as on the halting amplitude, and is given in supplementary eq. (4). To penalize the overlap $\langle 1|\psi\rangle_{\mathrm{anc}}$—which corresponds to wrong initialization, or halting—we add the following nearest neighbour term to the Hamiltonian:

$$\mathbf{h}^{(\mathrm{out})}_{i,i+1} = \Big|[\blacksquare][\circlearrowleft_1, \ldots, \xi]\Big\rangle\Big\langle[\blacksquare][\circlearrowleft_1, \ldots, \xi]\Big|_{i,i+1} \otimes (\mathbb{1}_i - |1\rangle\langle 1|_i) \otimes \mathbb{1}_{i+1}.$$

As just mentioned, the input penalty term $\mathbf{h}^{(\mathrm{in})}_{i,i+1}$ can similarly be written as a nearest-neighbour projector onto a clock state at $t = 0$. Thus, on the

entire chain we have the penalty terms

$$\mathbf{H}^{(\mathrm{in})} = \sum_{i=1}^{L-1} \mathbf{h}^{(\mathrm{in})}_{i,i+1} \tag{8}$$

$$\mathbf{H}^{(\mathrm{out})} = \sum_{i=1}^{L-1} \mathbf{h}^{(\mathrm{out})}_{i,i+1}. \tag{9}$$

Supplementary Definition 3.4. *We denote the QPE+QTM history state Hamiltonian including the in- and output penalties from supplementary eq.* (8) *with* $\mathbf{H}_{\mathrm{comp}}(L,\varphi) := \mathbf{H}_{\mathrm{QTM}}(L,\varphi) + \mathbf{H}^{(\mathrm{in})} + \mathbf{H}^{(\mathrm{out})}$.

3.3 Ground State Energy in Halting and Non-Halting Case

The ground state energy of $\mathbf{H}_{\mathrm{comp}}$ depends on how much penalty is picked up throughout the computation. Known techniques like Kitaev's geometrical lemma [KSV02; BCO17] for a lower bound and a simple triangle inequality for the upper bound can be used to show that

$$\Omega\left(\frac{1}{T^3}\right) \leq \lambda_{\min}(\mathbf{H}_{\mathrm{comp}}(\varphi(\eta))) \leq \mathrm{O}\left(\frac{1}{T}\right) \tag{10}$$

for a non-halting instance $\eta \in \mathbb{N}$. However, both the upper and lower bounds here are not tight enough for our purposes.

In order to obtain tighter bounds, we realize that our history state construction has a linear clock (i.e. one that never branches and simply runs from $t = 0$ to $t = T$); in this case, tight bounds on the overall energy effect of the penalty terms already exist; we refer the reader to [BC18; CLN18; Wat19] for an extended analysis. For the sake of completeness and brevity, we quote some of the definitions and lemmas from prior literature in the appendix and reference them in the following.

Supplementary Lemma 3.5. *In case* $\eta \in \mathbb{N}$ *correspond to a non-halting instance, the lowest eigenvalue of* $\mathbf{H}_{\mathrm{comp}}$ *satisfies* $\lambda_{\min}(\mathbf{H}_{\mathrm{comp}}(\varphi(\eta))) = \Omega(T^{-2})$.

Proof. In supplementary lemma 8.3, we prove $\mathbf{H}_{\mathrm{comp}}$ is a standard-form Hamiltonian as per supplementary definition 8.2, and so as per the Clairvoyance Lemma [Wat19, Lem. 5.6] we know that $\mathbf{H}_{\mathrm{comp}}$ breaks down into three subspaces. The subspaces of types 1 and 2 are trivially shown to have ground state energies $\Omega(T^{-2})$.

Within the third subspace, which we label S, there are no illegal terms and only the in- and output penalties $\mathbf{H}^{(\mathrm{in})} + \mathbf{H}^{(\mathrm{out})}$ from supplementary eq. (8) have to be considered. By supplementary lemma 8.6 the clock evolution within this subspace is linear—meaning there is never any branching—and

hence $\mathbf{H}_{\text{comp}}|_S$ is equivalent to Kitaev's original circuit-to-Hamiltonian construction. This means that the Hamiltonian therein is of the form

$$\mathbf{H}_{\text{comp}}|_S = \mathbf{H}_{\text{prop}} + |0\rangle\langle 0|_{\text{C}} \otimes \Pi^{(\text{in})} + |T\rangle\langle T|_{\text{C}} \otimes \Pi^{(\text{out})}$$

where $\mathbf{H}_{\text{prop}} \sim \Delta \otimes \mathbb{1}$ for a path graph Laplacian Δ, and $\Pi^{(\text{in})/(\text{out})}$ are the in- and output penalties inflicted at time 0 and T; this Hamiltonian is then explicitly of the family of Hamiltonians studied in [BC18]. In particular, by [BC18, Th. 7], Hamiltonians of this form have ground state energy $\lambda_{\min}(\mathbf{H}_{\text{comp}}|_S) = \Omega(T^{-2})$. Thus all three of the subspaces have a minimum eigenvalue of the form $\Omega(T^{-2})$, and since they are invariant subspaces, we see that the overall minimum eigenvalue must be $\lambda_{\min}(\mathbf{H}_{\text{comp}}) = \Omega(T^{-2})$. □

Supplementary Lemma 3.6 (Theorem 6.1 from [Wat19])**.** *Let $\mathbf{H}(\varphi) \in \mathcal{B}(\mathbb{C}^d)^{\otimes L}$ be a standard form Hamiltonian encoding a QTM with runtime $T(L)$, with in- and output penalty terms $\mathbf{H}^{(\text{in})/(\text{out})}$. Let there exist a computational path with no illegal states such that the final state of the computation is $|\psi_T\rangle$ and such that the output penalty term satisfies*

$$\langle T| \langle \psi_T| \mathbf{H}^{(\text{out})} |\psi_T\rangle |T\rangle \leq \epsilon.$$

Then the ground state energy is bounded by

$$0 \leq \lambda_{\min}\big(\mathbf{H}(\varphi)\big) \leq \epsilon \left(1 - \cos\left(\frac{\pi}{2(T - T_{\text{init}}) + 1}\right)\right) = \mathrm{O}\left(\frac{\epsilon}{T^2}\right),$$

where $T_{\text{init}} = \mathrm{O}(\log(T))$ is the time frame within which the input penalty term $\mathbf{H}^{(\text{in})}$ applies to the history state.

With this machinery developed, we can derive the following lemma for the specific Hamiltonian $\mathbf{H}_{\text{comp}}$ at hand.

Theorem 3.7. *Take $\mathbf{H}_{\text{comp}}$ to encode a phase $\varphi' \in [\varphi(\eta), \varphi(\eta) + 2^{-\ell})$, with $\varphi(\eta)$, as per supplementary definition 2.1, and let $\delta(L, m)$ be as in supplementary lemma 2.7. Then for*

1. *$m < \eta$ we have*
$$\lambda_{\min}(\mathbf{H}_{\text{comp}}) = \Omega\left[T^{-2}\right].$$

2. *$m \geq \eta$ and $\varphi(\eta)$ corresponds to a non-halting instance, then*
$$\lambda_{\min}(\mathbf{H}_{\text{comp}}) = \Omega\left[T^{-2}\right].$$

3. *$m \geq \eta$ and $\varphi(\eta)$ corresponds to a halting instance, then*
$$\lambda_{\min}(\mathbf{H}_{\text{comp}}) = \mathrm{O}\left[\left(2^{-\ell} + \delta(L, m)\right)^2 \frac{1}{T^2}\right].$$

Proof. Combing supplementary lemma 2.8 with supplementary lemma 3.1 we derive upper and lower bounds on the magnitude of the amplitudes that a given instance has on a non-halting state. Together with supplementary lemma 3.5 this gives us the lower bounds for points 1 and 2. To get the upper bound in 3, by supplementary lemma 2.8 and supplementary eq. (4), the output penalty is bounded as

$$\langle\psi_T|\,\Pi^{(\mathrm{out})}\,|\psi_T\rangle \le \sin^2\left(\frac{\pi}{3}\right)\left(2^{-\ell}+\delta(L,m)\right)^2.$$

Since no other term contributes a positive energy, the ground state of $\mathbf{H}_{\mathrm{comp}}$ can be upper-bounded with supplementary lemma 3.6 as

$$\lambda_{\min}(\mathbf{H}_{\mathrm{comp}}) = \mathrm{O}\left(\frac{\left(2^{-\ell}+\delta(L,m)\right)^2}{T^2}\right). \qquad \square$$

Supplementary Note 4 Checkerboard and TM Tiling

In the previous section we saw that the energy penalty from encoding a halting/non-halting computation in a Gottesman-Irani type Hamiltonian decreases as $\sim 1/T^2$ (see supplementary theorem 3.7). To find a way to boost this energy penalty such that it is no-longer dependent on T, we combine the Gottesman-Irani Hamiltonian with a classical Hamiltonian. Later we will partition the local Hilbert space into a classical part and quantum part $\mathcal{H}_c \otimes \mathcal{H}_q$, and choose interactions between the two parts of the Hilbert space to such that ground states of the Gottesman-Irani Hamiltonian are forced to be present at certain points in the tiling. We also encode a classical TM in the classical Hamiltonian, which will later be used to correct for errors in the approximate quantum phase estimation encoded in the Gottesman-Irani Hamiltonian.

4.1 Tiling to Hamiltonian Mapping

Given a fixed set of Wang tiles on a 2D lattice, we can map the corresponding tiling pattern to a classical translationally invariant, nearest neighbour Hamiltonian over spins on the same lattice. This is used to great effect in [GI09; CPGW15] and shown rigorously in lemma 1 and corollary 2 of [Bau+18a], where the authors also explain how to allow weighted tile sets. In the latter it is explained how to favour a certain tile by giving a bonus to it, or by giving an especially-strong penalty to a specific combination of tiles. For the sake of completeness, we will summarize the essence of the result below.

Let $\mathcal{T}$ be a set of Wang tiles. For an edge e in the interaction graph denote with $K \subset \mathcal{T} \times \mathcal{T}$ the subset of tiles that are allowed to be placed next to each other along edge e, and a function $w : K \longrightarrow \mathbb{R}$ assigns a weight

to a neighbouring tile pair. Then the corresponding local term is simply a weighted projector

$$\mathbf{h}^e = \sum_{(t_1,t_2)=t\in K} \big(\mathbb{1} - w(t)\,|t_1\rangle\langle t_1| \otimes |t_2\rangle\langle t_2|\,\big). \tag{11}$$

The overall Hamiltonian will then be a sum of these terms, i.e. $\mathbf{H} = \sum_e \mathbf{h}^e$, and its ground state is the highest-score tiling possible on the interaction graph. In the most simple case where $w \equiv 1$ this simply means that the ground state will have zero energy if there exists a tiling without a single mismatch anywhere that tiles the lattice. Its degeneracy will depend on how many tiling choices without any mismatching edges are possible.

It is obvious that when the original tiling constraints on the interaction graph were translationally-invariant, then so is the constructed Hamiltonian; furthermore, the local dimension of that Hamiltonian will equal the number of tiles that we need to allow per site.

In case we need to have more than one tile set on the same lattice, we can simply introduce lattice layers:

Supplementary Remark 4.1 (Tiling Layers [GI09]). *For multiple tile sets $\mathcal{T}_1, \ldots, \mathcal{T}_\ell$, there exists a meta tileset $\mathcal{T}$ with a set of meta-tiling rules, such that the meta-tiling rules are only satisfied iff the tiling rule for each element of the tuple is satisfied. The corresponding Hamiltonian is defined on the tensor product of the individual Hilbert spaces. Tile constraints may also be placed between layers.*

Proof. Given a lattice, we represent the meta-tile set as an ℓ-tuple associated with each site. Each element represents a layer in the tiling. Tiling rules for the kth layer are enforced between the kth elements of tuples on neighbouring sites. Tiling rules between layers can be prevented from occurring by disallowing certain tuples from appearing. □

4.2 Checkerboard Tiling

In this section, we define a tile set that periodically tiles the infinite plane. The underlying pattern we wish to create is that of a square lattice, where each grid cell within the pattern has the same side length, much like the boundaries on a checkerboard. The tiling will not be unique; in fact, there will be a countably infinite number of variants of the tiling which satisfy the tiling rules, corresponding to the pattern's periodicity. This non-uniquess is intended: the corresponding tiling Hamiltonian will have a degenerate ground state, the interplay of the other Hamiltonians' energy eigenstates that are conditioned on this underlying lattice pattern will then single out a unique ground state.

We constructively define this checkerboard tiling in this section. In order to explain and prove rigorously how the highest net-bonus tiling is achieved,

we break the proof up into two parts; in the first part, we will create a checkerboard pattern of various square sizes, but such that the offset from the lower left corner in the lattice is left unconstrained. In the second part, we will lift this degeneracy.

Supplementary Proposition 4.2 (Unconstrained Checkerboard Tiling)**.** *We define the tileset $\mathcal{T}_1$ to contain the following edge-colored tiles:*

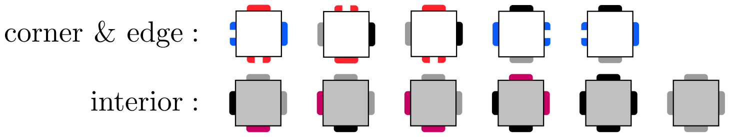

The rules for these tiles—by convention—are such that edges have to match up. Then all valid tilings for a lattice Λ will either:

1. *have no corner tile present, or*
2. *have corner tiles present as shown in supplementary fig. 3, i.e. such that they are part of a checkerboard pattern of squares, where the squares' side length—and the offset of the left- and bottommost corner tile—is unconstrained.*

Proof. Fig. 3 forms a valid tiling by inspection. What is left to prove is that given we demand at least one corner tile to be present this is the only tiling pattern possible.

To this end, we first note that the tiles directly adjacent to the corner tile are necessarily of the following configuration:

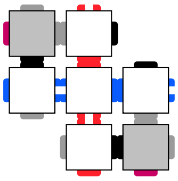

We then note that the only way for multiple of these corner tiles to join up is via blue horizontal links (called configuration A_2), red vertical ones (configuration A_3), or diagonal purple ones (configuration A_1); we show sections of these links A_1, A_2 and A_3 in supplementary fig. 2.

This reduces the problem to finding valid geometric patterns of horizontal blue, vertical red and diagonal purple lines, which are only ever allowed to intersect jointly together; the resulting pattern is a grid of squares laid out by the red and blue edge tiles, where the fact that each enclosed area is a square is enforced by the purple diagonals. If the square size is bigger than the lattice Λ, this means that only a single corner tile is present; otherwise there is multiple ones, as shown in supplementary fig. 3. Naturally, offset and square sizes remain unconstrained; the claim follows. □

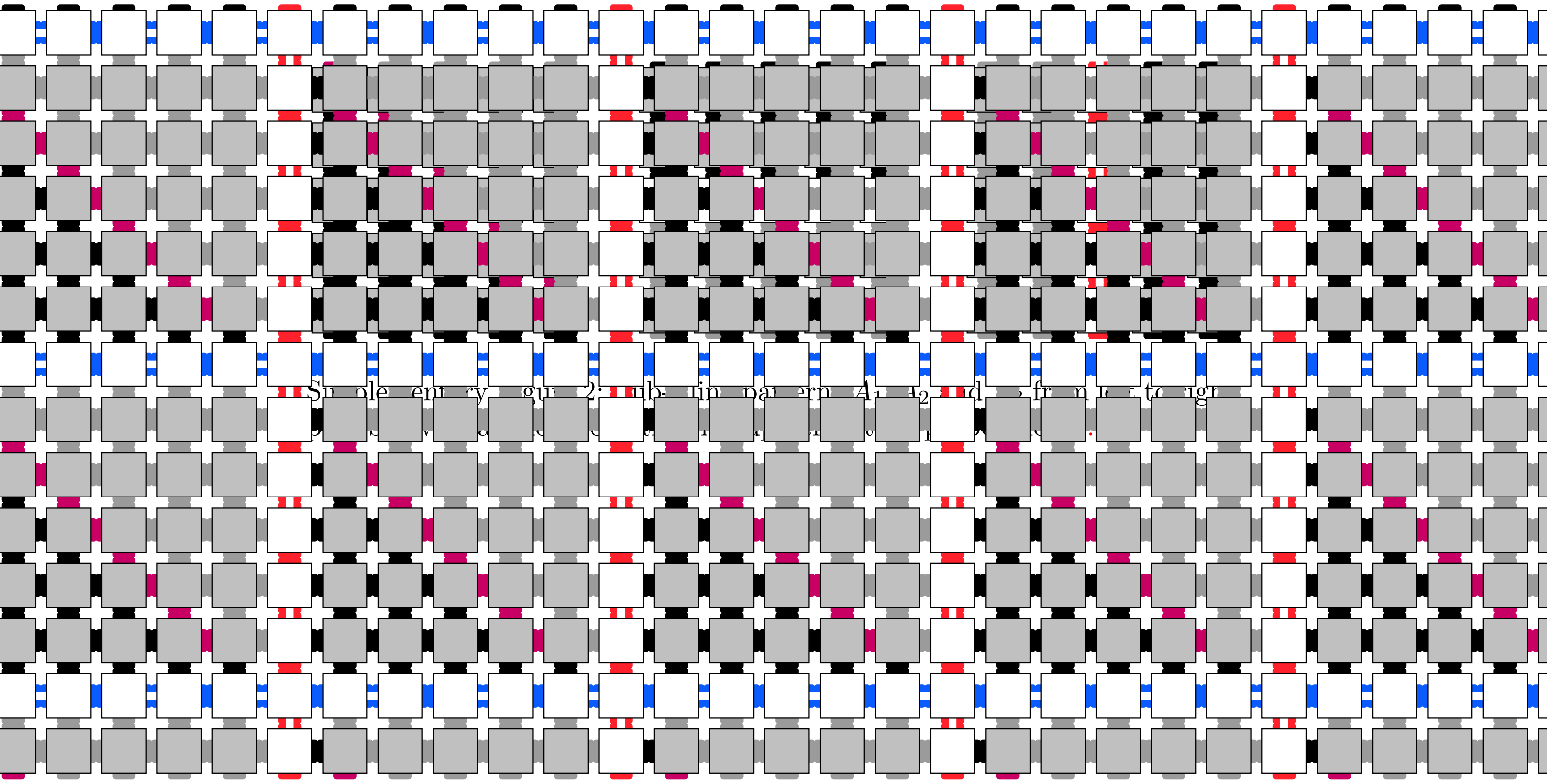

Supplementary Figure 3: Section of the checkerboard tiling Hamiltonian's ground state.

We emphasize that the tileset $\mathcal{T}$ in supplementary proposition 4.2 does have valid tilings that are e.g. all-grey- or all-black-edged areas, or those where only a purple diagonal with grey on one side, and black on the other side is present, as shown in supplementary fig. 2. For this reason, and in order to lift the offset degeneracy still present, we add extra constraints to the tileset.

In order to single out all those patterns that commence with a full square in the lower left corner of the lattice region, we employ Gottesman and Irani's boundary trick which exploits the fact that on any hyperlattice there is always a very specific mismatch between the number of vertices and the number of edges. In our case it reads as follows.

Supplementary Proposition 4.3 (Constrained Checkerboard Tiling)**.** *Take the tileset $\mathcal{T}$ from supplementary proposition 4.2 with the same edge-matching tiling rules, and define a new tileset $\mathcal{T}'$ with the following additional bonuses and penalties:*

1. *any interior tile gets a bonus of -1 if it appears to the top, and a bonus of -1 if it appears to the right of another tile, and*

2. *any interior tile gets an unconditional penalty of 2.*

Then the highest score tilings possible with $\mathcal{T}'$ on a square lattice Λ are the checkerboard patterns shown in supplementary fig. 3, but such that a corner tile lies in the lower left of the lattice. All other tilings have net score ≥ 1.

Proof. The only effect of the extra bonus and penalty terms are that the grey interior tiles can no longer appear on the left or bottom lattice boundaries; edge tiles have to be placed there. This, in turn, means that the only zero penalty configuration for the lower left corner is to place a corner tile there, meaning that the only net zero penalty configurations have at least one corner tile present. The rest of the claim then follows from supplementary proposition 4.2. □

With the tileset $\mathcal{T}'$ defined such that the highest net-score tilings are checkerboard patterns with unconstrained square sizes and offset $(0, 0)$ from the lower left corner in the spin lattice, we can formalize the tiling Hamiltonian in the following lemma.

Supplementary Lemma 4.4 (Checkerboard Tiling Hamiltonian)**.** *There exists a diagonal Hermitian operator $\mathbf{h} \in \mathcal{B}(\mathbb{C}^d \otimes \mathbb{C}^d)$ for $d = 11$ with matrix entries in $\mathbb{Z}$ as in supplementary eq. (11) such that the corresponding tiling Hamiltonian $\mathbf{H}_{\text{cb}} = \sum_{i\sim j} \mathbf{h}^{(i,j)}$ on a square lattice Λ has a degenerate zero energy ground space S_{cb} spanned by checkerboard tilings as in supplementary fig. 3, of all possible square sizes, where the pattern starts with a corner tile at the origin (i.e. in the lower left corner of the lattice), as laid out in supplementary proposition 4.3. Any other eigenstate not contained in this family of zero energy states has eigenvalue ≥ 1.*

Proof. Translating the tileset $\mathcal{T}'$ from supplementary proposition 4.3 into local terms as in supplementary eq. (11) via [Bau+18a] yields local Hamiltonian terms $\mathbf{h} \in \mathcal{B}(\mathbb{C}^d \otimes \mathbb{C}^d)$, where d is the number of tiles in the tileset—here 11; the local terms have entries in $\mathbb{Z}$ because all the weights (bonuses and penalties) in the tileset are integers. $\mathbf{H}_{\text{cb}}$ will have a ground space spanned by tilings with net score 0, which we proved in supplementary proposition 4.3 to look as claimed.

Furthermore, since all other tilings must have integer net penalty, all other tiling eigenstates will have energy ≥ 1. The claim follows. □

4.3 Classical Turing Machine Tiling

It is well know that a classical TM which runs for time N and uses a tape of length N can be encoded in an $N \times N$ grid of tiles [Ber66]. A brief overview of how this is done is given in the following. We first recall that a TM is specified by a tuple (Σ, Q, δ) where Q is the TM state, Σ is the TM alphabet, and δ is a transition function

$$\delta : Q \times \Sigma \to Q \times \Sigma \times \{L, R\}. \tag{12}$$

as well as an initial state q_0, an accepting state q_a, and a blank symbol $\# \in \Sigma$. Here L, R in the transition function output tell the TM head whether to move left or right respectively.

We now take the $N \times N$ grid which we can place tiles on. We will identify the rows of the grid with the tape of the TM, where successive rows will be successive time steps. Each tile now represents a cell of the TM's tape at a given time step. We now introduce a set of tiles which encode the evolution of the TM. We will need tiles which represents every possible configuration that a cell can take (what is written in the cell, whether the TM head is there, etc.).

To encode the evolution of such a TM into a set of tiles, we introduce three types of tiles: variety 1 which is specified only by an element of Σ, variety 2 specified by $\Sigma \times Q \times \{r, l\}$, and variety 3 specified by $\Sigma \times Q \times \{R, L\}$. At position P offset from the left within a row, these tiles have the following function:

Variety 1 With marking (c), $c \in \Sigma$, the corresponding cell on the TM's tape contains c, and the TM head is *not* at position P at the corresponding time step.

Variety 2 With marking (c, q, d), $c \in \Sigma$, $q \in Q$, $d \in \{r, l\}$, the corresponding cell on the TM's tape contains c, the TM head is at position P at this time step, but has not yet overwritten the tape symbol. The TM is in state q and the TM head has just moved from the right/left of P.

Variety 3 With entry (c, q, D), $c \in \Sigma$, $q \in Q$, $D \in \{R, L\}$, the corresponding cell on the TM's tape contains c, the TM head has just moved right/left from position P where it has just overwritten the previous symbol. The TM is in state q at this time step.

As a last remark, we note that one can always dovetail multiple TM tilings, as shown by Gottesman and Irani.

Supplementary Lemma 4.5 (Tiling-Layer Dovetailing [GI09])**.** *Let $\mathcal{M}_1$ and $\mathcal{M}_2$ be classical Turing machines with the same alphabet Σ such that their evolution is encoded in a tiling pattern on different tiling layers (see supplementary remark 4.1) of a rectangular grid with a border as in supplementary fig. 3. Then—by potentially altering the tile sets—it is possible to constrain the tiling layers at the border such that $\mathcal{M}_2$ takes the output of $\mathcal{M}_1$ as its input and continues the computation.*

Proof. If $\mathcal{M}_1$ and $\mathcal{M}_2$ are TMs, then there exists a TM M which carries out $\mathcal{M}_1$ followed by $\mathcal{M}_2$ [Tur37]. Define a tileset on each layer that corresponds to said Turing machine, such that $\mathcal{M}_1$ runs from bottom-to-top and $\mathcal{M}_2$ runs top-to-bottom on each respective layer. We now need to show that

there is a way of enforcing equality of the tapes of the two tiling layers next to the boundary; then the claim follows.

Similar to supplementary remark 4.1, let the meta tile at position k be specified by a 2-tuple $T_k = (t_i, t_j)_k$. Let the set of tiles making up the border be B. Then we enforce the 2-local tiling rule that the only valid tiles that can appear next to the upper border tiles have the form $((t_i, t_i), b)$, where $b \in B$ (i.e. the tiles must have the same markings in both layers). Thus the output of $\mathcal{M}_1$ is the input of $\mathcal{M}_2$. $\mathcal{M}_2$ then continues the computation on the top layer of the grid. □

In this fashion, any Turing machine (e.g. a universal one) can be encoded in a grid of tiles, which in turn can be used to define a local Hamiltonian with a ground state that corresponds to the TM's valid evolution; given a TM tiling, this can be achieved by using the tiling-to-Hamiltonian mapping already explained. Giving due credit, we capture this mapping for TM tilings in the following lemma.

Supplementary Lemma 4.6 (Berger's Turing machine Tiling Hamiltonian [Ber66]). *For any classical Turing machine (Σ, Q, δ) there exists a diagonal Hermitian operator $\mathbf{h} \in \mathcal{B}(\mathbb{C}^d \otimes \mathbb{C}^d)$ for $d = \mathrm{poly}(|\Sigma|, |Q|)$ with matrix entries in $\mathbb{Z}$ as in supplementary eq. (11) such that the corresponding tiling Hamiltonian $\sum_{i\sim j} \mathbf{h}^{(i,j)}$ on a square lattice Λ has a degenerate ground space $S_{\mathrm{TM,tiling}}$ containing*

1. *any tape configuration without TM head tiling the plane forward indefinitely,*
2. *a tiling pattern corresponding to valid Turing machine evolutions where the initial head is aligned on one side of the lattice and where the TM does not halt on the initial tape and space provided, and*
3. *any valid Turing machine evolution starting mid-way that does not halt within the space provided.*

Proof. See [Ber66]; the fact that the tape without head tiles the infinite plane is obvious since the tape can be initialized arbitrarily and will consistently cover the lattice, i.e. by being copied forwar. If the TM's head is present in a tile, and since there is no transition *into* the initial state $q_i \in Q$ of the TM, if the initial state is present it has to reside on one side of the lattice. Similarly, if the TM halts within the space provided there is no forward transition, meaning that tiling cannot have zero energy. Finally, if neither initial nor final state are present the tiling can show a consisten Turing machine evolution starting mid-way, with the tape being copied forward, or potentially altered if the TM head passes by. □

We emphasize that the TM in supplementary lemma 4.6 does not have to be reversible. We will later lift the large degeneracy of the so-defined ground

space by forcing an initial tape and head configuration; with such an initial setup, the tiling becomes unique since the forward evolution of a TM head and tape is always unambiguous.

4.4 Combining Checkerboard and Turing Machine Tiling

As seen in supplementary remark 4.1 and supplementary lemma 4.5, we can combine two tilesets into one, by defining the new tileset as the Cartesian product of the two. In this fashion we couple the TM tile set to apper above grey-shaded interior of the squares in the underlying checkerboard pattern from supplementary proposition 4.3; the area above the edge tiles we fill with a dummy border tile. We use this dummy border to enforce initialization of the TM's tape and head: for a tape cell *above but not to the right* of the border, the tape cell is blank. For a cell *above and to the right* of a border, we put the TM into its initial configuration q_0.

In case we need our Turing machine to run for more steps than are available on a single $L \times L$ grid, we can do so as well by introducing multiple layers as per supplementary remark 4.1.

Supplementary Lemma 4.7. *Let $n_1, n_2 \in \mathbb{N}$ be constant, and take a TM tileset $\mathcal{S}$ such that the TM tiles appear over the grey-shaded interior of the checkerboard pattern in supplementary fig. 3. We can define a new tileset $\mathcal{S}'$ such that the TM head will start in the lower left corner on an empty tape; on a grey square of side length L it will have a tape of length $n_1 L$ and runtime $n_2 L$ available.*

Proof. Initializing the head and tape on one edge of the grey square is achieved by penalizing any other tiles from appearing there, which we can do using inter-layer constraints as in supplementary remark 4.1. Once the TM tiling reaches one end of the grey square, we can similarly copy its state to another layer with a separate TM tileset that makes it evolve in the opposite direction. This shows that one can increase the available number of time steps by another constant n_2. An even simpler argument shows that on finitely many grid cells L one can always increase the number of tape cells by a constant factor n_1, by redefining n_1 sets of separate symbols. The claims follow. □

4.5 Cordonning off an Edge Subsection

In this section, we show that one can define a classical TM tiling that puts a single marker on an extra layer within each checkerboard square in supplementary fig. 3, namely on the lower edge, and at position $x = \lceil L^{1/c} \rceil$ for any $c \in \mathbb{N}$, $c > 0$. Since we have already shown how to define a classical TM tiling to appear only within the grey shaded interior of each checkerboard sqaure (supplementary remark 4.1), how to allow constant tape

and runtime overhead (supplementary lemma 4.7), and how to dovetail TM tilings (supplementary lemma 4.5), the claim is immediate from the following two lemmas.

Supplementary Lemma 4.8. *Let* $f : \mathbb{N} \to \mathbb{N}$ *such that* $1 \leq f(N) \leq N$ *be computable within time* $\mathrm{O}(2^{|x|})$ *where* $|x|$ *is the binary length of its input. Consider the checkerboard tiling constructed in supplementary proposition 4.3 such that each square has side length* $N+2$ *(which is measured between corner tiles). Then there exists a set of tiles which has this same checkerboard pattern, but for every corner tile, except those along the bottom edge, there is a special symbol* $\bullet$ *at distance* $f(N)$ *from the left border along the top edge.*

Proof. The proof is a variant of a construction from [GI09]. First, we add an extra tiling layer above the grey interior of the checkerboard tilings which translates the square's side length N into binary; this can be done with a counter tiling, see [GI09; Pat14] and [BP17, sec. F2.3].

Using supplementary lemma 4.5, we then dovetail this output with a TM that computes the function $f(N)$ by taking input from the previous layer. Since this is promised to be computable in time $\mathrm{O}(2^{|N|}) = \mathrm{O}(N)$, this can be done via supplementary lemma 4.7. The output of this computation is then $f(N)$ in binary.

Finally we run a binary-to-unary converting TM on the binary output of $f(N)$ by reversing the binary counter tiling in [GI09; Pat14]; this requires N steps. This leaves a marker at distance $f(N)$ along the square interior. We can then introduce a tiling rule which forces a $\bullet$ marker onto the edge above it. The configuration on the upper white edge of each complete square of the tiling is then

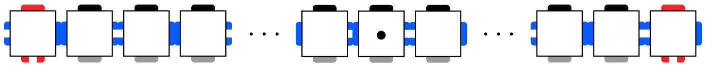

(13)

where the black dot $\bullet$ marks distance $f(N)$ away from the left border. □

With supplementary lemma 4.8 in place, all that is left is to show existence of a TM that calculates the 8th root of a number given in binary, and obeys the required constraint on the number of steps—i.e. at most linear in the square's side length L.

Supplementary Lemma 4.9. *Let* $c \in \mathbb{N}$, $c > 0$. *There exists a classical TM which, on binary input* L*, computes* $\lceil L^{1/8} \rceil$ *in binary, and requires at most* $\mathrm{O}(\log_2^8(L))$ *steps.*

Proof. It is known that taking the square root of a number has the same time complexity as multiplication (see [Alt79]). For a number of $\ell = \log_2 L$ digits, long multiplication has time- and space complexity $\sim \log_2^2 L$. Taking the 8th root can thus be done in $\sim \log_2^8 L$ steps by calculating

$$\sqrt[8]{\cdot} = \sqrt{\sqrt{\sqrt{\cdot}}}$$

□

Now we have all the ingredients together to define the following augmented checkerboard Hamiltonian, which is in essence the checkerboard tiling Hamiltonian defined in supplementary lemma 4.4, but with a classical TM acting within its grey squares to place an additional marker onto the horizontal edges.

Supplementary Lemma 4.10 (Augmented Checkerboard Tiling Hamiltonian)**.** *Let* $\mathbf{H}_{\text{cb}}$ *be the Hamiltonian defined in supplementary lemma 4.4. Then we can increase the local Hilbert space dimension to accommodate for the extra tileset necessary in supplementary lemma 4.9, and define a new Hamiltonian* $\mathbf{H}'_{\text{cb}}$ *as per supplementary eq.* (11) *where*

1. *the zero energy ground state is spanned by the same checkerboard patterns as in supplementary lemma 4.4, but such that the horizontal edges* above *a grey square carry a special marker* $\bullet$ *at offset* $L^{1/8}$ *from the left cornerstone,*

2. *any other eigenstate has eigenvalue* ≥ 1*.*

Proof. The first claim follows by supplementary lemmas 4.8 and 4.9, and supplementary lemma 4.6. The second claim follows since the grey TM interiors feature unique tilings, enforced by penalties only. □

For later reference, we further prove the following two tiling robustness facts.

Supplementary Remark 4.11 (Checkerboard Tiling Robustness)**.** *We single out the pair of tiles*

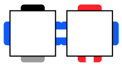

in the tileset $\mathcal{T}'$ *used in supplementary proposition 4.3. Then either*

1. *the pair of tiles is part of an edge of some length* L *as shown in the proof of supplementary lemma 4.8—i.e. fig. 13—with a grey square of size* $L \times L$ below *it, and a valid TM tiling enforcing the position of the extra edge marker* $\bullet$ *at position* $\lceil L^{1/8} \rceil$*, or*

2. *there exists a unique penalty* ≥ 1 *at another location in the lattice that can be associated to the tile pair.*

Proof. Since the corner tile in the pair cannot be one on the left lattice boundary, we follow the tiling to its left; it has to be a blue edge A_2 pattern as in supplementary fig. 2, and necessarily end in another corner tile—if not, take the mismatching tile and resulting penalty of size 1 as the unique associated one.

Given the blue horizontal edge is intact, this defines a distance between the two corner tiles, L. The subsquare $L \times L$ *below* this defined edge then has

to be a valid checkerboard square with augmenting TM as in supplementary lemma 4.10, which in turn enforces the position of the • marker between the two upper corner tiles at the specified offset. If the square is not intact—which includes it being cut off—take the closest penalty in Manhattan distance from the tile pair as the associated penalty of size ≥ 1 (or one of the closest one in case of ambiguities). ☐

Supplementary Remark 4.12 (Augmented Checkerboard Tiling Robustness)**.** *In any given ground state of the checkerboard tiling, there can be at most one • between two cornerstone markers; this marker is only ever present on blue horizontal edges that have a full grey interior square below them, meaning the • is offset at* $\lceil L^{1/8} \rceil$ *from its left, as in supplementary lemma 4.9. Any other configuration introduces a penalty* ≥ 1.

Proof. A bullet can only appear above the appropriate marker in the classical TM. We design the TM such that it produces exactly one such marker and such a marker gets a penalty if it is not above the point at which the TM places it. Thus, if there exists more than one • per edge joining two cornerstones, at most one of them can be above the marker left by the classical TM, and hence the other will receive an energy penalty.

Furthermore, since • can only occur at the output of a valid TM tiling, it can only occur on edges that lie above a full TM tiling. Since the lattice boundaries are white edges by supplementary proposition 4.3, the claim follows. ☐

Supplementary Note 5 A 2D Marker Checkerboard

In this section we will introduce a Hamiltonian on a one-dimensional spin chain which has a fine-tuned negative energy. More specifically, our goal in this section is to take the tiling pattern given in supplementary fig. 3 used to define $\mathbf{H}'_{\text{cb}}$ in supplementary lemma 4.10, and on a separate layer add the Marker Hamiltonian $\mathbf{H}^{(f)}$ from [Bau+18b, Thm. 11]. The Marker Hamiltonian, plus the classical TM encoded in the classical Hamiltonian will then be used to correct for the energy penalties caused by the quantum phase estimation being done approximately (and thus picking up some error).

5.1 Combining the Marker Hamiltonian with the Classical Tiling

In slight extension from the construction therein, we only allow the boundary markers $|\blacksquare\rangle$ to coincide with the cornerstones of the checkerboard tiling

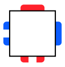

and condition the transition terms $\mathbf{h}_1$ and $\mathbf{h}_2$ from [Bau+18b, Lem. 2] to only occur in between two cornerstones and if and only if the marker • is present there,[1] i.e. on the blue horizontal edge

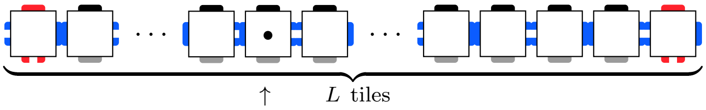

(14)

All other configurations are energetically penalised. The negative energy contribution of one such edge—and thus by supplementary remark 4.12 also of one square *below* said edge in the checkerboard pattern—is

$$E_{\text{edge}}(L) := \lambda_{\min}(\mathbf{H}^{(f)}|_{S(L)}), \tag{15}$$

where $S(L)$ denotes a single ■-bounded segment of the original marker construction of length L. The arrow ↑ denotes the position of the special symbol that indicates position $L^{1/8}$, as explained in supplementary lemmas 4.9 and 4.10.

As the ground state energy of $\mathbf{H}^{(f)}$ depends on the choice of the falloff f we carefully pick this function to be able to discriminate between the halting and non-halting cases in supplementary theorem 3.7. In particular, we will choose f such that if a universal TM halts on input $\varphi(\eta)$, then $\min_L(E_{\text{edge}}(L) + \lambda_{\min}(\mathbf{H}_{\text{comp}})) < 0$, if it does not halt then $\min_L(E_{\text{edge}}(L) + \lambda_{\min}(\mathbf{H}_{\text{comp}})) \geq 0$, where we assumed the Turing machine's tape length is L as well. For inputs $\varphi' \in [\varphi(\eta), \varphi(\eta) + 2^{-\eta-\ell})$ for some $\ell \geq 1$ a similar condition will be true depending on the amplitude that the output state has on halting and non-halting.

One obstacle is that the bounds on the energy contribution in [Bau+18b, Lem. 7] is too loose for our purposes, i.e. it was asymptotically bounded as lying in the interval $\lambda_{\min}(\mathbf{H}^{(f)}) \in (-2^{-f(L)}, -4^{-f(L)})$. In the following section, we prove that the scaling of the upper bound is in fact tight.

5.2 A Tight Marker Hamiltonian Bound

In this section we improve on the bounds set out in [Bau+18b, Lem. 7] for the ground state energy of the Marker Hamiltonian. To do this, we consider the following $w \times w$ matrix:

$$\Delta'_w = \Delta^{(w)} - |w\rangle\langle w| \,. \tag{16}$$

We now adapt [Bau+18b, Lem. 7] to prove a better lower bound on the lowest eigenvalue.

Supplementary Lemma 5.1. *The minimum eigenvalue of Δ'_w satisfies*

$$\lambda_{\min}(\Delta'_w) \geq -\frac{1}{2} - \frac{3}{4^w}. \tag{17}$$

[1] This can easily be enforced with a regular expression.

Proof. Our proof is essentially the same as in Bausch et al. except we use a better ansatz for the lower bound on the ground state energy. We begin by noting that, as in the proof of [Bau+18b, Lem. 7], the characteristic polynomial of Δ'_w is

$$p_w(\lambda) = -\frac{2^{-w-1}}{\sqrt{\lambda-4}}\left(3\sqrt{\lambda}(x_w(\lambda) - y_w(\lambda)) + \sqrt{\lambda-4}(x_w(\lambda) + y_w(\lambda))\right) \quad (18)$$

where

$$\begin{aligned} x_w(\lambda) &= \left(\lambda - \sqrt{\lambda-4}\sqrt{\lambda} - 2\right)^w \\ y_w(\lambda) &= \left(\lambda + \sqrt{\lambda-4}\sqrt{\lambda} - 2\right)^w . \end{aligned}$$

Since it is not clear if $p_w(\lambda) = 0$ has any closed form solutions in expressible in λ directly, we instead try to bound where the solutions can be.

First we calculate $p_w(-1/2) = (-1)^{1+w}2^{-w}$, and thus know that sgn $p_w(-1/2) = 1$ for w odd, and -1 for w even. If we can show that $p_w(-1/2 - f(w))$ has the opposite sign for some function $f(w) \geq 0$, then by the intermediate value theorem we know there has to exist a root in the interval $[-1/2 - f(w), -1/2]$. Since we are trying to prove a tighter bound than [Bau+18b, Lem. 7], we will assume $0 \leq f(w) \leq 2^{-w}$.

Let $p_w(-1/2 - f(w)) =: A_w/B_w$, where we use the notation of [Bau+18b, Lem. 7]:

$$\begin{aligned} B_w &= 2^{w+1}\sqrt{f(w) + \frac{9}{2}}, \\ A_w &= -a_{1,w}(x'_w - y'_w) - a_{2,w}(x'_w + y'_w), \\ a_{1,w} &= 3\sqrt{f(w) + \frac{1}{2}}, \\ a_{2,w} &= \sqrt{f(w) + \frac{9}{2}}, \\ x'_w &= \left(\sqrt{f(w) + \frac{9}{2}}\sqrt{f(w) + \frac{1}{2}} - f(w) - \frac{5}{2}\right)^w, \\ y'_w &= \left(-\sqrt{f(w) + \frac{9}{2}}\sqrt{f(w) + \frac{1}{2}} - f(w) - \frac{5}{2}\right)^w . \end{aligned}$$

Then B_w, $a_{1,w}$ and $a_{2,w}$ are real positive for all w. We distinguish two cases.

w even. If w is even, we need to show $p_w(-1/2 - 1/2^w) \geq 0$, which is equivalent to

$$
\begin{aligned}
& 0 \leq \frac{A_w}{B_w} \\
\iff\quad & 0 \leq A_w = -a_{1,w}(x'_w - y'_w) - a_{2,w}(x'_w + y'_w) \\
\iff\quad & 0 \geq a(x'_w - y'_w) + (x'_w + y'_w) \qquad \text{where } a := \frac{a_{1,w}}{a_{2,w}} \in [1,2] \\
\iff\quad & \frac{a-1}{a+1} y'_w \geq x'_w.
\end{aligned}
$$

For w even, $y'_w \geq x'_w$, and furthermore we find that $x'^{1/w}_w / y'^{1/w}_w$ is monotonically decreasing (assuming that $f(w) \geq 0$ and is itself monotonically decreasing), so it suffices to find a $f(w)$ which satisfies

$$\frac{a-1}{a+1} \geq \left(\frac{5}{2} - \frac{3}{2}\right)^w \left(\frac{5}{2} + \frac{3}{2}\right)^{-w} = \frac{1}{4^w}. \tag{19}$$

Expanding out a as

$$a = 3\sqrt{\frac{f(w) + 1/2}{f(w) + 9/2}}, \tag{20}$$

and substituting this into the above, we find

$$f(w) \geq \frac{9}{4(4^w) - 10 + 5(4^{-w})}. \tag{21}$$

Hence we can choose $f(w) = 3/4^w$, which works for all $w \geq 2$.

w odd. Now $y'_w \leq x'_w$, and it suffices to show

$$\frac{a-1}{a+1} y'_w \leq x'_w$$

which is true provided

$$\frac{a-1}{a+1} \leq 1.$$

This also holds true for all $w \geq 0$ for $f(w) = 3/4^w$. This finishes the proof. ☐

Theorem 5.2. *The minimum eigenvalue of Δ'_w satisfies*

$$-\frac{1}{2} - \frac{3}{4^w} \leq \lambda_{\min}(\Delta'_w) \leq -\frac{1}{2} - \frac{1}{4^w}. \tag{22}$$

Proof. Supplementary Lemma 5.1 gives the lower bound, and [Bau+18b, Lem. 8] gives the upper bound. ☐

5.3 Balancing QPE Error and True Halting Penalty

With this tighter bound derived in supplementary theorem 5.2, we can calculate the necessary magnitude and scaling of $E_{\text{edge}}(L)$ as explained at the start of supplementary note 5 as follows. As a first step, we notice that the clock runtime $T = T(L)$ of the QTM is bounded by supplementary eq. (7), which holds both in the halting and non-halting case, since the clock idles after the computation is done. That is, the clock runtime *does not* depend on the input to the computation.

Let $E_{\text{pen,halt}}(L)$ and $E_{\text{pen,too short}}(L)$ be the ground state energies of $\mathbf{H}_{\text{comp}}(L)$ in the case where the encoded computation does not halt with high probability, and when the binary expansion of the encoded phase is too long, respectively, i.e. when $|\varphi'| > m$. Then from supplementary theorem 3.7 we get:

$$E_{\text{pen,non-halt}}(L) \geq E_{\text{pen,too short}}(L) = \Omega\left[\frac{1}{T^2}\right] \overset{*}{\geq} \frac{K_1}{L^2 \xi^{2L} \log^2 L}, \tag{23}$$

where we made use of supplementary remark 2.9 at step $(*)$. Similarly, let $E_{\text{pen,halt}}(L)$ be the minimum eigenvalue when the QTM halts on input $\varphi' \in [\varphi(\eta), \varphi(\eta) + 2^{-\eta-\ell})$, as given in supplementary definition 2.1. Then again from supplementary theorem 3.7 and for sufficiently large ℓ we get:

$$\begin{aligned} E_{\text{pen,halt}}(L) &= \mathrm{O}\left[\left(2^{-\ell} + \delta(L,m)\right)\frac{1}{T^2}\right] \\ &\overset{**}{=} \mathrm{O}\left[\left(2^{-\ell} + L^2 2^{-L^{1/4}}\right)\frac{1}{T^2}\right] \leq \frac{K_2 2^{-L^{1/4}}}{\xi^{2L}}. \end{aligned} \tag{24}$$

where in step $(**)$ we have used the fact that $m \leq L$, $c_1 < 4$ and $c_2 \geq 1$. Both K_1 and K_2 in supplementary eqs. (23) and (24) are positive constants, chosen sufficiently small and large to satisfy the two bounds. How large does ℓ have to be—or in other words, how small does the interval around $\varphi(\eta)$ have to be that φ' is chosen from—for supplementary eq. (24) to hold?

$$2^{-\ell} \leq L^2 2^{-L^{1/4}} \quad \Leftrightarrow \quad \ell \geq \log_2\left(L^{-2} 2^{L^{1/4}}\right). \tag{25}$$

In order to discriminate between the two asymptotic history state penalties in supplementary eqs. (23) and (24), $E_{\text{edge}}(L)$ thus has to lie asymptotically between these two bounds, i.e. we need

$$E_{\text{edge}}(L) = \mathrm{o}\left(\frac{1}{L^2 \xi^{2L} \log^2 L}\right) \quad \text{and} \quad E_{\text{edge}}(L) = \omega\left(\frac{1}{\xi^{2L} 2^{L^{1/4}}}\right).$$

Now we know by supplementary theorem 5.2 that $E_{\text{edge}}(L) \sim 4^{-f(L)}$ for some $f : \mathbb{N} \longrightarrow \mathbb{N}$ marker falloff, which itself has to be computable by a

history state construction on the segment of length L. We therefore require

$$\mathrm{o}\left(\frac{1}{L^2\xi^{2L}\log^2 L}\right) = \frac{1}{4^{f(L)}} = \omega\left(\frac{1}{\xi^{2L}2^{L^{1/4}}}\right), \quad \text{or}$$
$$\omega\left(L+\log L+\log\log L\right) = f(L) = \mathrm{o}\left(L+L^{1/4}\right). \tag{26}$$

This lets us formulate the following conclusion.

Supplementary Corollary 5.3. *There exists a constant C such that $f(L) = C(L+L^{1/8})$ asymptotically satisfies supplementary eq. (26).*

5.4 Marker Hamiltonian with $\mathbf{L + L^{1/8}}$ Falloff

The crucial question is: can we create a Marker Hamiltonian with a falloff exponent like $f(L) = C(L+L^{1/8})$, which would satisfy supplementary corollary 5.3? As discussed in [Bau+18b], this is certainly possible for any polynomial of L, or even an exponential—in essence it is a question of creating another history state clock for which the runtime of the segment of length L equals $f(L)$. Herein lies the problem: while a runtime L is easy—just have a superposition of a particle sweeping from one side to the other—how do we perform $L^{1/8}$ additional steps?

While there might be a clever way of doing this purely within the scope of a history state construction, we take the easy way out.[2] In supplementary note 4.5, we discussed how we can place a special symbol on the lower edge, which by supplementary lemma 4.9 can be at distance $L^{1/8}$ from the left corner. With this in mind and with the tighter marker Hamiltonian spectral bound from supplementary lemma 5.1 to define the following variant of a marker Hamiltonian:

Supplementary Lemma 5.4. *Let $C \in \mathbb{N}$ be constant. Take the standard marker Hamiltonian $\mathbf{H}_0^{(f)}$ from [Bau+18b] defined on a local Hilbert space $\mathcal{H}_0 = \mathbb{C}^{d'}$, where d' depends on the decay function f to be implemented. Then there exists a variant $\mathbf{H}^{(f)}$ with local Hilbert space $\mathcal{H} = \mathcal{H}_0 \otimes \mathbb{C}^2$, where $|\bigstar\rangle$ is one of the basis states of the second subspace, such that $\mathbf{H}^{(f)}$ has the following additional properties:*

1. *$\mathbf{H}^{(f)} = \sum_i \mathbf{h}_i$, with $\mathbf{h}_i \in \mathcal{B}(\mathbb{C}^d \otimes \mathbb{C}^d)$, and $d = \mathrm{O}(C)$.*
2. *$[\mathbf{h}, |\bigstar\rangle\langle\bigstar|] = 0$.*
3. *If $S(r)$ is the subspace of a single $\blacksquare$-bounded segment of length L, containing a single $\bigstar$ offset at position r, then*

$$-\frac{3}{4^{f(L)}} \le \lambda_{\min}\left(\mathbf{H}^{(f)}|_{S(r)}\right) \le -\frac{1}{4^{f(L)}}, \tag{27}$$

[2] We note that if this task is possible within the history state framework, then it may be possible to prove the main result of this paper for 1D. Indeed, the 2D tiling construction is only used to allow the 1D Marker Hamiltonian to have the correct drop off.

where $f(L) = C(L + r)$*.*

Proof. We design the marker Hamiltonian variant to perform the following procedure before stopping:

1. Sweep the length of the edge L,
2. Sweep back to the $|\bigstar\rangle$ symbol sitting at offset r.
3. If the number of rounds is not yet C, switch to another head state and repeat, where even iterations run in reverse.

Finally, employ Gottesman and Irani's boundary trick, used as in [Bau+18b, Rem. 3], which exploits the mismatch in number of one- and two-local interaction terms to remove the constant $-1/2$ offset present in supplementary theorem 5.2 by only adding translationally-invariant nearest neighbour terms to the Hamiltonian. The energy scaling then follows directly from supplementary theorem 5.2, and the dimension and $[\mathbf{h}_M, |\bigstar\rangle\langle\bigstar|] = 0$ follow by construction. □

This marker Hamiltonian we will now combine with the Hilbert space of the checkerboard Hamiltonian $\mathbf{H}'_{\mathrm{cb}}$ from supplementary lemma 4.10, to obtain a 1D marker Hamiltonian where the location of the boundary symbols ■ and offset marker ★ align with the checkerboard tiles as

$\blacksquare \longleftrightarrow$ 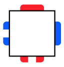 and $\bigstar \longleftrightarrow$ 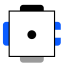 (28)

and such that the marker Hamiltonian terms do not occur above any other but the blue edge tiles.

Supplementary Corollary 5.5 (1D Marker Hamiltonian)**.** *Let* $\mathbf{H}'_{\mathrm{cb}}$ *be the checkerboard Hamiltonian from supplementary lemma 4.10, with local Hilbert space* $\mathcal{H}_{\mathrm{cb}}$*. Take* $\mathbf{H}^{(f)}$ *from supplementary lemma 5.4, with local Hilbert space* $\mathcal{H}$*, and let* $C \in \mathbb{N}$*,* $C \geq 1$*. Then there exists a marker Hamiltonian* $\mathbf{H}_1^{(f)}$ *with one- and two-local interactions* $\mathbf{h}_1 \in \mathcal{B}(\mathcal{H}')$*,* $\mathbf{h}_2 \in \mathcal{B}(\mathcal{H}' \otimes \mathcal{H}')$ *where* $\mathcal{H}' := (\mathcal{H} \oplus \mathbb{C}) \otimes \mathcal{H}_{\mathrm{cb}}$*, and such that* $\mathbf{H}_1^{(f)}$ *has the following properties.*

1. *If* $S(r)$ *denotes the subspace of a good tiling edge segment supplementary eq. (13) of length* L*, where the marker* • *is offset at position* r *from the left, then*
$$-\frac{3}{4^{f(L)}} \leq \lambda_{\min}\left(\mathbf{H}_1^{(f)}|_{S(r)}\right) \leq -\frac{1}{4^{f(L)}},$$
with $f(L) = C(L + r)$*.*
2. *Restricted to any other tiling subspace* S' *which does not contain the pair of tiles*

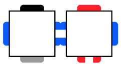

we have $\lambda_{\min}(\mathbf{H}_1^{(f)}|_{S'}) \geq 0$*.*

Proof. Let $\mathbf{h}_1'$ and $\mathbf{h}_2'$ denote the one- and two-local terms of $\mathbf{H}^{(f)}$, trivially extended to the larger Hilbert space $\mathcal{H} \oplus \mathbb{C}$. Let $|0\rangle$ denote the extra basis state in $\mathcal{H} \oplus \mathbb{C}$. Denote with Π a projector onto the tiling subspace spanned by the corner and blue edge tiles given in supplementary proposition 4.2. We explicitly construct the local interactions $\mathbf{h}_1$ and $\mathbf{h}_2$ of $\mathbf{H}_1^{(f)}$ by setting

$$\mathbf{h}_1 := \mathbf{h}_1' \otimes \Pi + |0\rangle\langle 0| \otimes \Pi + (\mathbb{1} - |0\rangle\langle 0|) \otimes \Pi^{\perp} +$$
$$(\mathbb{1} - |\bigstar\rangle\langle\bigstar|) \otimes \Big|$$ 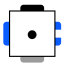 $$\Big\rangle\Big\langle$$ 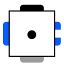 $$\Big| + (\mathbb{1} - |\blacksquare\rangle\langle\blacksquare|) \otimes \Big|$$ 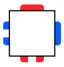 $$\Big\rangle\Big\langle$$ 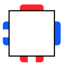 $$\Big|$$

and

$$\mathbf{h}_2 := \mathbf{h}_2' \otimes \Pi^{\otimes 2}.$$

The marker bonus is only ever picked up by the (final state) marker head running into the right boundary in a configuration $|\cdots \triangleright\triangleright\blacktriangleright\blacksquare\rangle$, which by the one-local Hamiltonian constraints newly imposed can only occur above the tile pair blue edge–corner given; any other configuration will have a net penalty ≥ 0. By construction, the ground space of $\mathbf{H}_1^{(f)}$ features the required alignment from supplementary eq. (28). The claim then follows from supplementary lemma 5.4. $\square$

This is the last ingredient we require to formulate a two-dimensional variant of the Marker Hamiltonian, with the required falloff from supplementary corollary 5.3.

Theorem 5.6 (2D Marker Hamiltonian)**.** *We denote with Λ the given lattice. Let $\mathbf{h}_1$ and $\mathbf{h}_2$ be the local terms defining the 1D marker Hamiltonian from supplementary corollary 5.5 with constant $C \in \mathbb{N}$, $C \geq 1$. Further let $\mathbf{H}_{\mathrm{cb}}'$ be the augmented checkerboard lattice with symbol $\bullet$ offset by $L^{1/8}$ on each of the horizontal edges, as defined in supplementary lemma 4.10. On the joint Hilbert space we set*

$$\mathbf{H}^{(\boxplus,f)} := \mathbb{1} \otimes \mathbf{H}_{\mathrm{cb}}' + \sum_{i\in\Lambda} \mathbf{h}_1^{(i)} + \sum_{i\in\Lambda} \mathbf{h}_2^{(i)}$$

where the second sum runs over any grid index where the 2×1-sized interaction can be placed. Then the following hold:

1. *$\mathbf{H}^{(\boxplus,f)}$ block-decomposes as $\mathbf{H}^{(\boxplus,f)} = \bigoplus_{s=1}^{L} \mathbf{H}_s^{(\boxplus,f)} \oplus \mathbf{B}$; the family $\mathbf{H}_s^{(\boxplus,f)}$ corresponds to all those tiling patterns compatible with the augmented checkerboard pattern in supplementary lemma 4.10 with square size s. $\mathbf{B}$ collects all other tiling configurations.*

2. *The ground state of $\mathbf{H}_s^{(\boxplus,f)}$, labelled $|\psi_s\rangle$ is product across squares $|\psi_s\rangle = \bigotimes_i |\phi_i\rangle$, where i runs over all squares in the tiling.*

3. $\mathbf{B} \geq 0$.

4. *Denote with A a single square of the ground state $|\boxplus_s\rangle$ (i.e. a square making up the grid), denoted $|\boxplus_s\rangle_A$. Then its energy contribution to the ground state of $\mathbf{H}_s^{(\boxplus,f)}$ is*

$$-\frac{3}{4^{C(s+s^{1/8})}} \leq \langle\boxplus_s|_A \, \mathbf{H}^{(\boxplus,f)}(s)|_A \, |\boxplus_s\rangle_A \leq -\frac{1}{4^{C(s+s^{1/8})}}.$$

where C is the constant from supplementary corollary 5.5.

5. *Denote with $\Pi = |\boxplus_s\rangle\langle\boxplus_s|_A$ the projector onto the orthogonal complement of the ground state of $\mathbf{H}_s^{(\boxplus,f)}|_A$. Then*

$$\Pi \mathbf{H}_s^{(\boxplus,f)}|_A \Pi \geq 0.$$

Proof. We prove the claims step by step.

Claim 1 & 2 The classical tiling Hamiltonian $\mathbf{H}'_{\text{cb}}$ is diagonal in the computational basis. Furthermore, by construction, $\mathbf{h}_1$ and $\mathbf{h}_2$ defined in supplementary corollary 5.5 commute with the tiling terms.

Claim 3 The bonus of $-1/2$ introduced in the marker Hamiltonian can only ever act across a pair of tiles

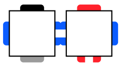

Since we have proven the checkerboard tiling to be robust with respect to the occurence of this tile pair in supplementary remark 4.11, we know that the combination carries at least a penalty ≥ 1 if it occurs in any non-checkerboard configuration; this means that any tiling in $\mathbf{B}$ can never have a sub-configuration such that the marker bonus offsets penalties inflicted by the tiling constraints; $\mathbf{B} \geq 0$ follows.

Claim 4 The "good" subspace in the fourth claim we know by supplementary remark 4.12 to necessarily look as the blue edge segment supplementary eq. (13). This, in turn, means that $r = \lceil L^{1/8} \rceil$ in supplementary lemma 5.4, and the claim follows from the first energy bound proven therein.

Claim 5 Follows in a similar fashion as the fourth claim, from supplementary remark 4.12 and from the second claim in supplementary lemma 5.4. □

Supplementary Note 6 Spectral Gap Undecidability of a Continuous Family of Hamiltonians

In this section, we combine the 2D Marker Hamiltonian with the QPE History State construction. Despite the two-dimensional marker Hamiltonian, the setup is very reminiscent of the 1D construction; the crucial difference being the more finely-geared bonus and penalties we need to analyse. We show that the energy contributions from each of the checkerboard squares, defined in the classical Hamiltonian, is either positive or negative depending on whether an encoded computation halts or not. This provides a constant ground state energy density which is either positive or negative which can be leveraged to prove undecidability of the spectral gap and phase.

6.1 Uncomputability of the Ground State Energy Density

Supplementary Lemma 6.1. *Let* $\mathbf{h}_1, \mathbf{h}_2^{\mathrm{row}}, \mathbf{h}_2^{\mathrm{col}}$ *be the one- and two-local terms of* $\mathbf{H}^{(\boxplus,f)}$ *with local Hilbert space* $\mathcal{H}_{\mathrm{m}}$*, and similarly denote with* $\mathbf{q}_1, \mathbf{q}_2$ *be the one- and two-local terms of* $\mathbf{H}_{\mathrm{comp}}$ *from supplementary definition 3.4 with local Hilbert space* $\mathcal{H}_{\mathrm{q}}$*, respectively. Let* Π_{edge} *be a projector onto the edge tiles in supplementary proposition 4.2. Define the combined Hilbert space* $\mathcal{H} := \mathcal{H}_{\mathrm{m}} \otimes (\mathcal{H}_{\mathrm{q}} \oplus \mathbb{C})$*, where* $|0\rangle$ *denotes the basis state for the extension of* $\mathcal{H}_{\mathrm{q}}$*.*

We define the following one- and two-local interactions:

$$\mathbf{h}_1^{\mathrm{tot}} := \mathbf{h}_1 \otimes \mathbb{1} + \Pi_{\mathrm{edge}} \otimes \mathbf{q}_1 + \Pi_{\mathrm{edge}} \otimes |0\rangle\langle 0| + \Pi_{\mathrm{edge}}^{\perp} \otimes (1 - |0\rangle\langle 0|)$$

$$\mathbf{h}_2^{\mathrm{tot,row}} := \mathbf{h}_2^{\mathrm{row}} \otimes \mathbb{1} + \Pi_{\mathrm{edge}}^{\otimes 2} \otimes \mathbf{q}_2$$

$$\mathbf{h}_2^{\mathrm{tot,col}} := \mathbf{h}_2^{\mathrm{col}} \otimes \mathbb{1}$$

$$\mathbf{p}_2^{\mathrm{tot,row}} :=$$

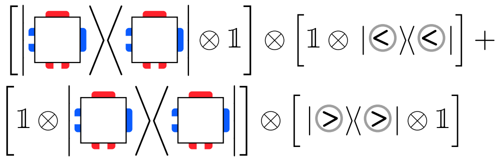

On a lattice Λ *define the overall Hamiltonian*

$$\mathbf{H} := \sum_{i \in \Lambda} \mathbf{h}_{1,(i)}^{\mathrm{tot}} + \sum_{i \in \Lambda} \left(\mathbf{h}_{2,(i)}^{\mathrm{tot,row}} + \mathbf{p}_{2,(i)}^{\mathrm{tot,row}} \right) + \sum_{i \in \Lambda} \mathbf{h}_{2,(i)}^{\mathrm{tot,col}},$$

where each sum index runs over the lattice Λ *where the corresponding Hamiltonian term can be placed. Then* $\mathbf{H}$ *has the following properties:*

1. $\mathbf{H} = \bigoplus_s \mathbf{H}_s \oplus \mathbf{B}'$ *block-decomposes as* $\mathbf{H}^{(\boxplus,f)}$ *in supplementary theorem 5.6, where* $\mathbf{B}' = \mathbf{B} \otimes \mathbb{1}$*.*

2. $\mathbf{B}' \geq 0$*.*

3. *All eigenstates of* $\mathbf{H}_s$ *are product states across squares in the tiling with square size* s*, product across rows within each square, and product across the local Hilbert space* $\mathcal{H}_\mathrm{m} \otimes (\mathcal{H}_\mathrm{q} \oplus \mathbb{C})$.

4. *Within a single square* A *of side length* s *within a block* $\mathbf{H}_s$*, all eigenstates are of the form* $|\boxplus_s\rangle\,|_A \otimes |r_0\rangle \otimes |r\rangle$*, where*

   (a) $|\boxplus_s\rangle$ *is the ground state of the 2D marker Hamiltonian block* $\mathbf{H}_s^{(\boxplus,f)}$,

   (b) $|r_0\rangle$ *is an eigenstate of* $\mathbf{H}_\mathrm{comp} \oplus \mathbf{0}$*, i.e. the history state Hamiltonian with local padded Hilbert space* $\mathcal{H}_\mathrm{q} \oplus \mathbb{C}$*, and*

   (c) $|r\rangle \in (\mathcal{H}_\mathrm{q} \oplus \mathbb{C})^{\otimes(s\times(s-1))}$ *defines the state elsewhere.*

5. *The ground state of* $\mathbf{H}_s|_A$ *is unique and given by* $|r\rangle = |0\rangle^{\otimes(s\times(s-1))}$ *and* $|r_0\rangle = |\Psi\rangle$*, where*

$$|\Psi\rangle = \sum_{t=0}^{T-1} |t\rangle\,|\psi_t\rangle$$

   is the history state of $\mathbf{H}_\mathrm{comp}$ *as per supplementary theorem 3.3, and such that* $|\psi_0\rangle$ *is correctly initialized.*

Proof. We already have all the machinery in place to swiftly prove this lemma. First note that, by construction, all of $\{\mathbf{h}_1^\mathrm{tot}, \mathbf{h}_2^\mathrm{tot,row}, \mathbf{h}_2^\mathrm{tot,col}, \mathbf{h}_2^\mathrm{col}, \mathbf{p}_2^\mathrm{tot,row}\}$ pairwise commute with the respective tiling Hamiltonian terms $\{\mathbf{h}_1, \mathbf{h}_2^\mathrm{row}, \mathbf{h}_2^\mathrm{col}\}$. Furthermore, the local terms from $\mathbf{H}_\mathrm{comp}$—$\mathbf{q}_1$ and $\mathbf{q}_2$—are positive semi-definite; together with supplementary theorem 5.6 this proves the first three claims. As shown in supplementary theorem 3.3 and since the Hamiltonian constraints in $\mathbf{p}_2^\mathrm{tot,row}$ enforce the ground state of the top row within the square A to be bracketed, the first and third claim imply the fourth and fifth. □

Supplementary Lemma 6.2. *Take the same setup as in supplementary lemma 6.1, and let* $\mathbf{H}_\mathrm{comp} = \mathbf{H}_\mathrm{comp}(\varphi')$ *for* $\varphi' \in [\varphi(\eta), \varphi(\eta) + 2^{-\eta-\ell})$*, where* $\varphi(\eta)$ *is the unary encoding of* $\eta \in \mathbb{N}$ *from supplementary definition 2.1. As usual* $\ell \geq 1$*. Then for a block* $\mathbf{H}_s$ *we have*

1. *If* $s < \eta$, $\mathbf{H}_s \geq 0$.

2. *If* $s \geq \eta$ *and* $\mathcal{M}$ *does not halt on input* η *within space* s*, then* $\mathbf{H}_s \geq 0$.

3. *If* $s \geq \eta$ *and* $\mathcal{M}$ *halting on input* η*, and* $\ell \geq \log_2(s^{-2}2^{s^{1/4}})$ *as per supplementary eq. (25), then* $\lambda_\mathrm{min}(\mathbf{H}_s) < 0$.

Proof. We start with the first claim. By supplementary lemma 6.1, it suffices to analyse a single square A of side length s; the proof then essentially follows that of [Bau+18b, Thm. 20]. We first assume $s < \eta$. Using the same notation

as in supplementary theorem 5.6, and denoting with Π_{edge} the projector onto the white horizontal edge within A, we have

$$\begin{aligned}\lambda_{\min}(\mathbf{H}_s|_A) &= \lambda_{\min}\left[\mathbf{H}^{(\boxplus,f)}(s)|_A \otimes \mathbb{1} + \Pi_{\text{edge}} \otimes \mathbf{H}_{\text{comp}}(\varphi')\right] \\ &= E_{\text{edge}}(s) + E_{\text{pen,tooshort}}(s) \geq 0,\end{aligned}$$

where we used supplementary corollary 5.3 and supplementary theorem 5.6 and the fact that the two Hamiltonian terms in the sum commute.

The other claims follow equivalently: in each case by supplementary corollary 5.3, the sum of the edge bonus and TM penalties satisfy supplementary eq. (26). For the second claim, by the same process we thus get

$$\lambda_{\min}(\mathbf{H}_s|_A) = E_{\text{edge}}(s) + E_{\text{pen,non-halt}}(s) \geq 0.$$

Then for the third claim,

$$\lambda_{\min}(\mathbf{H}_s|_A) = E_{\text{edge}}(s) + E_{\text{pen,halt}}(s) < 0. \qquad \square$$

Supplementary Corollary 6.3. *Take the same setup as in supplementary lemma 6.2, and let $\varphi(\eta)$ encode a halting instance. Set $w = \operatorname{argmin}_s\{\lambda_{\min}(\mathbf{H}_s) < 0\}$, and W a single square of size $w \times w$. Then the ground state energy of $\mathbf{H}(\varphi')$ on a grid Λ of size $L \times H$ is bounded as*

$$\lambda_{\min}(\mathbf{H}(\varphi')) = \left\lfloor \frac{L}{w} \right\rfloor \left\lfloor \frac{H}{w} \right\rfloor \lambda_{\min}(\mathbf{H}(\varphi')|_W). \tag{29}$$

Proof. From supplementary lemma 6.1, we know the ground state of $\mathbf{H}(\varphi')$ is a grid with offset $(0,0)$ from the lattice's origin in the lower left. Each square of the grid contributes energy $\lambda_{\min}(\mathbf{H}(\varphi')|_W) < 0$; the prefactor in supplementary eq. (29) is simply the number of complete squares within the lattice.

For all truncated squares on the right hand side, $\mathbf{H}_{\text{comp}}$ from supplementary definition 3.4 with either the left or right ends truncated has zero ground state energy, since it is either free of the in- or output penalty terms. Furthermore, we see that if we truncate the right end of the 1D Marker Hamiltonian $\mathbf{H}_1^{(f)}$ in supplementary lemma 5.4, it has a zero energy ground state since it never encounters the tile pair

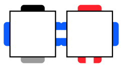

from supplementary theorem 5.6 necessary for a bonus. Truncating squares at the top does not yield any positive or negative energy contribution. The total lattice energy is therefore simply the number of complete squares on the lattice, multiplied by the energy contribution of each square. $\square$

Theorem 6.4 (Undecidability of Ground State Energy Density)**.** *Discriminating between a negative or nonnegative ground state energy density of* $\mathbf{H}(\varphi')$ *is undecidable.*

Proof. Immediate from supplementary lemma 6.2 and supplementary corollary 6.3; the energy of a single square is either a small negative constant, or nonnegative. Determining which is at least as hard as solving the halting problem. □

With this result we can almost lift the undecidability of ground state energy density to the spectral gap problem. In order to make the result slightly stronger, for this we first shift the energy of $\mathbf{H}(\varphi')$ by a constant.

Supplementary Lemma 6.5 ([Bau+18b, Lem. 23])**.** *By adding at most two-local identity terms, we can shift the energy of* $\mathbf{H}$ *from supplementary lemma 6.1 such that*

$$\lambda_{\min}(\mathbf{H}) \begin{cases} \geq 1 & \textit{in the non-halting case, and} \\ \longrightarrow -\infty & \textit{otherwise.} \end{cases}$$

6.2 Undecidability of the Spectral Gap

With the proven uncomputability of the ground state energy density, we can lift the result using the usual ingredients—a Hamiltonian with a trivial ground state, as well as a dense spectrum Hamiltonian that will be pulled down alongside the spectrum of the QPE Hamiltonian, if the encoded universal Turing machine halts on the input encoded in the phase parameter—to prove that the existence of a spectral gap for our constructed one-parameter family of Hamiltonians is undecidable as well.

Theorem 6.6 (Undecidability of the Spectral Gap)**.** *For a continuous-parameter family of Hamiltonians, discriminating between gapped with trivial ground state* $|0\rangle^{\otimes\Lambda}$*, and gapless as defined in supplementary definitions 1.1 and 1.2, is undecidable.*

Proof. So far we have constructed a Hamiltonian $\mathbf{H}(\varphi')$ with undecidable ground state energy asymptotics given in supplementary lemma 6.5; we denote its Hilbert space with $\mathcal{H}_1$. We add the usual Hamiltonian ingredients as in [CPGW15] or [Bau+18b, Thm. 25]:

$\mathbf{H}_{\text{dense}}$ Asymptotically dense spectrum in $[0, \infty)$ on Hilbert space $\mathcal{H}_2$.

$\mathbf{H}_{\text{trivial}}$ Diagonal in the computational basis, with a single 0 energy product ground state $|0\rangle^{\otimes\Lambda}$, and a spectral gap of 1 (i.e. all other eigenstates have nonnegative energy ≥ 0); its Hilbert space we denote with $\mathcal{H}_3$.

$\mathbf{H}_{\text{guard}}$ A 2-local Ising type interaction on $\mathcal{H} := \mathcal{H}_1 \otimes \mathcal{H}_2 \oplus \mathcal{H}_3$ defined as

$$\mathbf{H}_{\text{guard}} := \sum_{i \sim j} \left(\mathbb{1}_{1,2}^{(i)} \otimes \mathbb{1}_3^{(j)} + \mathbb{1}_3^{(i)} \otimes \mathbb{1}_{1,2}^{(j)} \right),$$

where the summation runs over all neighbouring spin sites of the underlying lattice Λ (horizontal and vertical).

We then define

$$\mathbf{H}^{\Lambda(L)}(\varphi') := \mathbf{H}(\varphi') \otimes \mathbb{1}_2 \oplus \mathbf{0}_3 + \mathbb{1}_1 \otimes \mathbf{H}_{\text{dense}} \oplus \mathbf{0}_3 + 0_{1,2} \oplus \mathbf{H}_{\text{trivial}} + \mathbf{H}_{\text{guard}}.$$

The guard Hamiltonian ensures that any state with overlap both with $\mathcal{H}_1 \otimes \mathcal{H}_2$ and $\mathcal{H}_3$ will incur a penalty ≥ 1. It is then straightforward to check that the spectrum of $\mathbf{H}_{\text{tot}}$ is given by

$$\operatorname{spec}(\mathbf{H}^\Lambda) = \{0\} \cup (\operatorname{spec}(\mathbf{H}(\varphi')) + \operatorname{spec}(\mathbf{H}_{\text{dense}})) \cup G$$

for some $G \subset [1, \infty)$, where the single zero energy eigenstate stems from $\mathbf{H}_{\text{trivial}}$.

In case that $\lambda_{\min}(\mathbf{H}(\varphi')) \geq 1$, $\operatorname{spec}(\mathbf{H}(\varphi')) + \operatorname{spec}(\mathbf{H}_{\text{dense}}) \subset [1, \infty)$ and hence the ground state of $\mathbf{H}^\Lambda$ is the ground state of $\mathbf{H}_{\text{trivial}}$ with a spectral gap of size one.

For $\lambda_{\min}(\mathbf{H}(\varphi')) \longrightarrow -\infty$, $\mathbf{H}_{\text{dense}}$ is asymptotically gapless and dense; this means that $\mathbf{H}^\Lambda$ becomes asymptotically gapless as well. □

Since the spectral properties of $\mathbf{H}(\varphi')$ are—by supplementary lemma 6.2—robust to a choice of φ' within an interval around an encoded instance $\varphi(\eta)$ as per supplementary definition 2.1—i.e. for large enough ℓ we can vary $\varphi' \in [\varphi(\eta), \varphi(\eta) + 2^{-\eta-\ell})$—supplementary theorem 6.6 immediately proves theorem 2.1, corollary 2.2 and corollary 2.3 in the main article.

As a small addition, we generalise the above to show undecidability between any two phases with an arbitrary ground state property.

Supplementary Corollary 6.7. *Let there exist Hamiltonians $\mathbf{H}_X$ and $\mathbf{H}_{\neg X}$ defined by local terms that their ground states respectively have the property X and do not have the property X, for all $L \geq L_0$ for some L_0, and both have zero ground state energy. Without loss of generality, let the ground state of $\mathbf{H}(\varphi')$ have the property X. For a continuous-parameter family of Hamiltonians, discriminating between a phase with property X, and a phase without ground state property X, is undecidable.*

Proof. We then define

$$\mathbf{H}^{\Lambda(L)}(\varphi') := \mathbf{H}(\varphi') \otimes \mathbb{1}_2 \oplus \mathbf{0}_3 + \mathbb{1}_1 \otimes \mathbf{H}_X \oplus \mathbf{0}_3 + 0_{1,2} \oplus \mathbf{H}_{\neg X} + \mathbf{H}_{\text{guard}}.$$

If $\lambda_0(\mathbf{H}(\varphi')) \to -\infty$, the ground state of $\mathbf{H}^{\Lambda(L)}(\varphi')$ has property X. If $\lambda_0(\mathbf{H}(\varphi')) \geq 1$, the ground state does not have property X. Since the ground state energy of $\mathbf{H}(\varphi')$ is undecidable, then determining whether the ground state has property X is also undecidable. □

6.3 Subtleties Concerning Computable and Uncomputable Numbers

In the statement of the main result and throughout the analysis, we have been careful to avoid a subtle point related to allowing φ to vary over all of $\mathbb{R}$: a number chosen uniformly from $\mathbb{R}$ is almost surely uncomputable. For such uncomputable values of φ, we cannot even write out the matrix elements of the local terms of the Hamiltonian. Thus determining the phase of the Hamiltonian is trivially uncomputable for such values.

However, it is worth noting that this is not an issue unique to the Hamiltonian constructed in this work; *any* Hamiltonian which is a function of parameters in $\mathbb{R}$ will suffer from this. For example, take the 1D Ising model with Hamiltonian $\mathbf{H}_{\text{TIM}}(\varphi) = \sum_{\langle i,j \rangle} \sigma_z^{(i)} \sigma_z^{(j)} + \varphi \sum_i \sigma_x^{(i)}$ for $\varphi \in \mathbb{R}$. The matrix elements for the local terms become uncomputable when φ is uncomputable. Despite this, the phase diagram for the model is computable [Sac11], and for all computable numbers φ the phase of $\mathbf{H}_{\text{TIM}}(\varphi)$ can be determined.

Our results are non-trivial because they hold even for *computable* values of $\varphi \in \mathbb{R}$.

Supplementary Note 7 Conclusions

One of the main aims of this work was as a first foray into the rigorous study of the computability and computational complexity of phase transitions. Quantum phase transitions are one of the best studied physical phenomena, but still incompletely understood. We anticipate that this work can be extended in several directions:

Uncomputablity in 1D. Here we have only studied phase diagrams in 2D. As described in 5, our construction has relied on the fact we can encode a classical Turing Machine into 2D tilings. This is not possible in 1D. However, since 1D systems tend to be fundamentally easier to solve than 2D systems, it may still be the case that the phase diagram of a 1D system is computable. However, given the undecidability of the spectral gap in 1D [Bau+18b], it would not be unexpected that computing phase diagrams of 1D systems can also be shown to be uncomputable.

More Realistic Systems. This work has shown that determining phase diagrams is in general uncomputable. But does this remain the case for any physically realistic systems—for example those with smaller Hilbert space dimension or interactions limited to a certain form?

Finite Systems. In this work we have only studied phase diagrams in the thermodynamic limit. Yet for any finite-sized system, determining any

property is necessarily decidable (as we can simply diagonalise the Hamiltonian). A natural question is thus what we can say about the complexity of determining phases and phase parameters for finite system sizes, for a suitable notion of phase transition in this context.

Supplementary Note Acknowledgements

J. B. would like to thank the Draper's Research Fellowship at Pembroke College. J. D. W. is supported by the EPSRC Centre for Doctoral Training in Delivering Quantum Technologies. T. S. C. is supported by the Royal Society. This work was supported by the EPSRC Prosperity Partnership in Quantum Software for Simulation and Modelling (EP/S005021/1).

Supplementary Note References

[Alt79] H. Alt. "Square rooting is as difficult as multiplication". *Computing* 21.3 (1979), pp. 221–232.

[BC18] Johannes Bausch and Elizabeth Crosson. "Analysis and limitations of modified circuit-to-Hamiltonian constructions". *Quantum* 2 (2018), p. 94. arXiv: 1609.08571.

[Bau+18a] Johannes Bausch, Toby S. Cubitt, Angelo Lucia, David Perez-Garcia, and Michael M. Wolf. "Size-driven quantum phase transitions". *Proceedings of the National Academy of Sciences* 115.1 (2018), pp. 19–23. arXiv: 1512.05687.

[Bau+18b] Johannes Bausch, Toby Cubitt, Angelo Lucia, and David Perez-Garcia. "Undecidability of the Spectral Gap in One Dimension" (2018). arXiv: 1810.01858.

[BCO17] Johannes Bausch, Toby Cubitt, and Maris Ozols. "The Complexity of Translationally-Invariant Spin Chains with Low Local Dimension". *Annales Henri Poincaré* (2017), p. 52. arXiv: 1605.01718.

[BP17] Johannes Bausch and Stephen Piddock. "The complexity of translationally invariant low-dimensional spin lattices in 3D". *Journal of Mathematical Physics* 58.11 (2017), p. 111901. arXiv: 1702.08830.

[Ber66] Robert Berger. *The Undecidability of the Domino Problem*. American Mathematical Soc., 1966, p. 72.

[BV97] Ethan Bernstein and Umesh Vazirani. "Quantum Complexity Theory". *SIAM Journal on Computing* 26.5 (1997), pp. 1411–1473.

[CLN18] Libor Caha, Zeph Landau, and Daniel Nagaj. "Clocks in Feynman's computer and Kitaev's local Hamiltonian: Bias, gaps, idling, and pulse tuning". *Physical Review A* 97.6 (2018), p. 062306. arXiv: 1712.07395.

[CPGW15] T. S. Cubitt, D. Perez-Garcia, and M. M. Wolf. *Undecidability of the spectral gap*. 2015. arXiv: 1502.04573 [quant-ph].

[DN05a] Christopher M. Dawson and Michael A. Nielsen. "The Solovay-Kitaev algorithm" (2005). arXiv: 0505030 [quant-ph].

[DN05b] Christopher M. Dawson and Michael A. Nielsen. "The Solovay-Kitaev algorithm" (2005). arXiv: 0505030 [quant-ph].

[GI09] Daniel Gottesman and Sandy Irani. "The quantum and classical complexity of translationally invariant tiling and Hamiltonian problems". *Foundations of Computer Science, 2009. FOCS'09. 50th Annual IEEE Symposium on*. IEEE. 2009, pp. 95–104.

[KSV02] Alexei Yu. Kitaev, Alexander Shen, and Mikhail N. Vyalyi. "Classical and quantum computing". *Quantum Information*. New York, NY: Springer New York, 2002, pp. 203–217.

[NC10] Michael A. Nielsen and Isaac L. Chuang. *Quantum Computation and Quantum Information*. Cambridge: Cambridge University Press, 2010, p. 676.

[Pat14] Matthew J. Patitz. "An introduction to tile-based self-assembly and a survey of recent results". *Natural Computing* 13.2 (2014), pp. 195–224.

[Sac11] Subir Sachdev. *Quantum Phase Transitions*. 2nd ed. Cambridge University Press, 2011.

[Tur37] A. M. Turing. "On Computable Numbers, with an Application to the Entscheidungsproblem". *Proceedings of the London Mathematical Society* s2-42.1 (1937), 230–265.

[Wat19] James D. Watson. "Detailed Analysis of Circuit-to-Hamiltonian Mappings". *arXiv e-prints*, arXiv:1910.01481 (Oct. 2019), arXiv:1910.01481. arXiv: 1910.01481 [quant-ph].

Supplementary Note 8 Standard Form Hamiltonians

We begin with the following definition for a 1D chain of spins:

Supplementary Definition 8.1 (Standard Basis States, from Section 4.1 of [CPGW15]). *Let the single site Hilbert space be $\mathcal{H} = \otimes_i \mathcal{H}_i$ and fix some orthonormal basis for the single site Hilbert space. Then a* Standard Basis State *for $\mathcal{H}^{\otimes L}$ are product states over the single site basis.*

We now define standard-form Hamiltonians – extending the definition from [CPGW15]:

Supplementary Definition 8.2 (Standard-form Hamiltonian, from [Wat19], extended from [CPGW15])**.** *We say that a Hamiltonian $H = H_{trans} + H_{pen} + H_{in} + H_{out}$ acting on a Hilbert space $\mathcal{H} = (\mathbb{C}^C \otimes \mathbb{C}^Q)^{\otimes L} = (\mathbb{C}^C)^{\otimes L} \otimes (\mathbb{C}^Q)^{\otimes L} =: \mathcal{H}_C \otimes \mathcal{H}_Q$ is of standard form if $H_{trans,pen,in,out} = \sum_{i=1}^{L-1} h^{(i,i+1)}_{trans,pen,in,out}$, and $h_{trans,pen,in,out}$ satisfy the following conditions:*

1. *$h_{trans} \in \mathcal{B}\left((\mathbb{C}^C \otimes \mathbb{C}^Q)^{\otimes 2}\right)$ is a sum of transition rule terms, where all the transition rules act diagonally on $\mathbb{C}^C \otimes \mathbb{C}^C$ in the following sense. Given standard basis states $a, b, c, d \in \mathbb{C}^C$, exactly one of the following holds:*
   - *there is no transition from ab to cd at all; or*
   - *$a, b, c, d \in \mathbb{C}^C$ and there exists a unitary U_{abcd} acting on $\mathbb{C}^Q \otimes \mathbb{C}^Q$ together with an orthonormal basis $\{\left|\psi^i_{abcd}\right\rangle\}_i$ for $\mathbb{C}^Q \otimes \mathbb{C}^Q$, both depending only on a, b, c, d, such that the transition rules from ab to cd appearing in h_{trans} are exactly $|ab\rangle \left|\psi^i_{abcd}\right\rangle \to |cd\rangle U_{abcd} \left|\psi^i_{abcd}\right\rangle$ for all i. There is then a corresponding term in the Hamiltonian of the form $(|cd\rangle \otimes U_{abcd} - |ab\rangle)(\langle cd| \otimes U^\dagger_{abcd} - \langle ab|)$.*

2. *$h_{pen} \in \mathcal{B}\left((\mathbb{C}^C \otimes \mathbb{C}^Q)^{\otimes 2}\right)$ is a sum of penalty terms which act non-trivially only on $(\mathbb{C}^C)^{\otimes 2}$ and are diagonal in the standard basis, such that $h_{pen} = \sum_{(ab)\ Illegal} |ab\rangle_C \langle ab| \otimes \mathbb{1}_Q$, where (ab) are members of a disallowed/illegal subspace.*

3. *$h_{in} = \sum_{ab} |ab\rangle \langle ab|_C \otimes \Pi_{ab}$, where $|ab\rangle \langle ab|_C \in (\mathbb{C}^C)^{\otimes 2}$ is a projector onto $(\mathbb{C}^C)^{\otimes 2}$ basis states, and $\Pi^{(in)}_{ab} \in (\mathbb{C}^Q)^{\otimes 2}$ are orthogonal projectors onto $(\mathbb{C}^Q)^{\otimes 2}$ basis states.*

4. *$h_{out} = |xy\rangle \langle xy|_C \otimes \Pi_{xy}$, where $|xy\rangle \langle xy|_C \in (\mathbb{C}^C)^{\otimes 2}$ is a projector onto $(\mathbb{C}^C)^{\otimes 2}$ basis states, and $\Pi^{(in)}_{xy} \in (\mathbb{C}^Q)^{\otimes 2}$ are orthogonal projectors onto $(\mathbb{C}^Q)^{\otimes 2}$ basis states.*

We note that although h_{out} and h_{in} have essentially the same form, they will play a conceptually different role.

Supplementary Lemma 8.3. **$\mathbf{H}_{\mathrm{QTM}}$** *is a standard form Hamiltonian.*

Proof. Comparing with supplementary definition 8.2, we see that all terms fall into one of the four classifications, and hence it is standard form. □

We now introduce the following definition.

Supplementary Definition 8.4 (Legal and Illegal Pairs and States, from [CPGW15]). *The pair ab is an* illegal pair *if the penalty term* $|ab\rangle\langle ab|_C \otimes \mathbb{1}_Q$ *is in the support of the* H_{pen} *component of the Hamiltonian. If a pair is not illegal, it is legal. We call a standard basis state* legal *if it does not contain any illegal pairs, and illegal otherwise.*

Then the following is a straightforward extension of Lemma 42 of [CPGW15] with H_{in} and H_{out} terms included.

Supplementary Lemma 8.5 (Invariant subspaces, extended from Lemma 42 of [CPGW15]). *Let* H_{trans}*,* H_{pen}*,* H_{in} *and* H_{out} *define a standard-form Hamiltonian as defined in supplementary definition 8.2. Let* $\mathcal{S} = \{S_i\}$ *be a partition of the standard basis states of* $\mathcal{H}_C$ *into minimal subsets* S_i *that are closed under the transition rules (where a transition rule* $|ab\rangle_{CD}|\psi\rangle \rightarrow |cd\rangle_{CD} U_{abcd}|\psi\rangle$ *acts on* $\mathcal{H}_C$ *by restriction to* $(\mathbb{C}^C)^{\otimes 2}$*, i.e. it acts as* $ab \rightarrow cd$*). Then* $\mathcal{H} = (\bigoplus_S \mathcal{K}_{S_i}) \otimes \mathcal{H}_Q$ *decomposes into invariant subspaces* $\mathcal{K}_{S_i} \otimes \mathcal{H}_Q$ *of* $H = H_{pen} + H_{trans} + H_{in} + H_{out}$ *where* $\mathcal{K}_{S_i}$ *is spanned by* S_i*.*

Supplementary Lemma 8.6 (Clairvoyance Lemma, extended from Lemma 43 of [CPGW15]). *Let* $H = H_{trans} + H_{pen} + H_{in} + H_{out}$ *be a standard-form Hamiltonian, as defined in supplementary definition 8.2, and let* $\mathcal{K}_S$ *be defined as in Lemma 8.5. Let* $\lambda_0(\mathcal{K}_S)$ *denote the minimum eigenvalue of the restriction* $H|_{\mathcal{K}_S \otimes \mathcal{H}_Q}$ *of* $H = H_{trans} + H_{pen} + H_{in} + H_{out}$ *to the invariant subspace* $\mathcal{K}_S \otimes \mathcal{H}_Q$*.*

Assume that there exists a subset $\mathcal{W}$ *of standard basis states for* $\mathcal{H}_C$ *with the following properties:*

1. *All legal standard basis states for* $\mathcal{H}_C$ *are contained in* $\mathcal{W}$*.*
2. $\mathcal{W}$ *is closed with respect to the transition rules.*
3. *At most one transition rule applies in each direction to any state in* $\mathcal{W}$*. Furthermore, there exists an ordering on the states in each S such that the forwards transition (if it exists) is from* $|t\rangle \rightarrow |t+1\rangle$ *and the backwards transition (if it exists) is* $|t\rangle \rightarrow |t-1\rangle$*.*
4. *For any subset* $S \subseteq \mathcal{W}$ *that contains only legal states, there exists at least one state to which no backwards transition applies and one state to which no forwards transition applies. Furthermore, the unitaries associated with the transition* $|t\rangle \rightarrow |t+1\rangle$ *are* $U_t = \mathbb{1}_Q$*, for* $0 \leq t \leq T_{init} - 1$ *and* $T_{init} < T$*, and that the final state* $|T\rangle$ *is detectable by a 2-local projector acting only on nearest neighbour qudits.*

Then each subspace $\mathcal{K}_S$ *falls into one of the following categories:*

1. *S contains only illegal states, and* $H|_{\mathcal{K}_S \otimes \mathcal{H}_Q} \geq \mathbb{1}$*.*

2. *S contains both legal and illegal states, and*

$$W^\dagger H|_{\mathcal{K}_S\otimes\mathcal{H}_Q} W \geq \bigoplus_i \big(\Delta^{(|S|)} + \sum_{|k\rangle\in K_i} |k\rangle\langle k|\big) \tag{30}$$

where $\sum_{|k\rangle\in K_i} |k\rangle\langle k| := H_{pen}|_{\mathcal{K}_S\otimes\mathcal{H}_Q}$ *and* K_i *is some non-empty set of basis states and* W *is some unitary.*

3. *S contains only legal states, then there exists a unitary* $R = W(\not{\mathbb{1}}_C \otimes (X\oplus Y)_Q)$ *that puts* $H|_{\mathcal{K}_S\otimes\mathcal{H}_Q}$ *in the form*

$$R^\dagger H|_{\mathcal{K}_S\otimes\mathcal{H}_Q} R = \begin{pmatrix} H_{aa} & H_{ab} \\ H_{ab}^\dagger & H_{bb} \end{pmatrix}, \tag{31}$$

where, defining $G := \operatorname{supp}\left(\sum_{t=0}^{T_{init}-1} \Pi_t^{(in)}\right)$ *and* $s := \dim G$*,*

- $X : G \to G$.
- $Y : G^c \to G^c$.
- H_{aa} *is an* $s\times s$ *matrix.*
- $H_{aa}, H_{bb} \geq 0$ *and are rank* r_a, r_b *respectively.*
- H_{aa} *has the form*

$$H_{aa} = \bigoplus_i \big(\Delta^{(|S|)} + \alpha_i \,||S|-1\rangle\langle|S|-1|\big) + \sum_{t=0}^{T_{init-1}} |t\rangle\langle t| \otimes X^\dagger \Pi_t|_G X. \tag{32}$$

- H_{bb} *is a tridiagonal, stoquastic matrix of the form*

$$H_{bb} = \bigoplus_i (\Delta^{(|S|)} + \beta_i \,||S|-1\rangle\langle|S|-1|). \tag{33}$$

- $H_{ab} = H_{ba}$ *is a real, negative diagonal matrix with rank* $\min\{r_a, r_b\}$.

$$H_{ab} = H_{ba} = \bigoplus_i \gamma_i \,||S|-1\rangle\langle|S|-1|\,. \tag{34}$$

where either we get pairings between the blocks such that

$$\begin{pmatrix} \alpha_i & \gamma_i \\ \gamma_i & \beta_i \end{pmatrix} = \begin{pmatrix} 1-\mu_i & -\sqrt{\mu_i(1-\mu_i)} \\ -\sqrt{\mu_i(1-\mu_i)} & \mu_i \end{pmatrix} \quad or \quad \begin{pmatrix} 1 & 0 \\ 0 & 1 \end{pmatrix}, \tag{35}$$

for $0 \leq \mu_i \leq 1$*, or we get unpaired values of* $\alpha_i = 0, 1$ *or* $\beta_i = 0, 1$ *for which we have no associated value of* γ_i*.*
